# Modeled Sea Level Rise Impacts on Coastal Ecosystems at Six Major Estuaries on Florida’s Gulf Coast: Implications for Adaptation Planning

**DOI:** 10.1371/journal.pone.0132079

**Published:** 2015-07-24

**Authors:** Laura L. Geselbracht, Kathleen Freeman, Anne P. Birch, Jorge Brenner, Doria R. Gordon

**Affiliations:** 1 The Nature Conservancy, Florida Chapter, Altamonte Springs, Florida, United States of America; 2 The Nature Conservancy, Texas Chapter, Corpus Christi, Texas, United States of America; 3 Department of Biology, University of Florida, Gainesville, Florida, United States of America; University of Vigo, SPAIN

## Abstract

The Sea Level Affecting Marshes Model (SLAMM) was applied at six major estuaries along Florida’s Gulf Coast (Pensacola Bay, St. Andrews/Choctawhatchee Bays, Apalachicola Bay, Southern Big Bend, Tampa Bay and Charlotte Harbor) to provide quantitative and spatial information on how coastal ecosystems may change with sea level rise (SLR) and to identify how this information can be used to inform adaption planning. High resolution LiDAR-derived elevation data was utilized under three SLR scenarios: 0.7 m, 1 m and 2 m through the year 2100 and uncertainty analyses were conducted on selected input parameters at three sites. Results indicate that the extent, spatial orientation and relative composition of coastal ecosystems at the study areas may substantially change with SLR. Under the 1 m SLR scenario, total predicted impacts for all study areas indicate that coastal forest (-69,308 ha; -18%), undeveloped dry land (-28,444 ha; -2%) and tidal flat (-25,556 ha; -47%) will likely face the greatest loss in cover by the year 2100. The largest potential gains in cover were predicted for saltmarsh (+32,922 ha; +88%), transitional saltmarsh (+23,645 ha; na) and mangrove forest (+12,583 ha; +40%). The Charlotte Harbor and Tampa Bay study areas were predicted to experience the greatest net loss in coastal wetlands The uncertainty analyses revealed low to moderate changes in results when some numerical SLAMM input parameters were varied highlighting the value of collecting long-term sedimentation, accretion and erosion data to improve SLAMM precision. The changes predicted by SLAMM will affect exposure of adjacent human communities to coastal hazards and ecosystem functions potentially resulting in impacts to property values, infrastructure investment and insurance rates. The results and process presented here can be used as a guide for communities vulnerable to SLR to identify and prioritize adaptation strategies that slow and/or accommodate the changes underway.

## Introduction

Coastal wetland systems and human communities will be substantially affected whether global sea level rises 0.18–0.59 m by 2100, as estimated by the IPCC (2007) [1], or at the higher rates based on more recent information and modeling [2, 3, 4, 5, 6]. In Florida, local sea level rise has generally mimicked global mean sea level rise of approximately 3 mm per year since 1993 although there has been some variation along Florida’s Gulf Coast (1.6 to 3.19 mm per year) as calculated from several decades of tide gauge records [7]. Coastal ecosystems are the foundation for the quality of life and the economies of many people across the globe [8, 9, 10, 11, 12]. Sea level rise may substantially change coastal ecosystems globally by increasing dry-land loss due to submergence and erosion, wetland loss and change, flood damage, saltwater intrusion into surface and ground water, and by raising water tables and impeding drainage [13]. Coastal ecosystems such as beaches, dunes, barrier islands and marshes may be able to migrate landward as sea level rises, but if development or other impediments are in the way, these systems will be squeezed and lose spatial extent [14]. Rocky shorelines will erode more quickly in the face of sea level rise [15]. Some coral reefs appear to be able to keep up with current rates of sea level rise, but this may vary regionally due to variations in the local rates of sea level rise [16]. Coastal lagoons may suffer changes in circulation, tidal exchange and turbidity with sea level rise and may also shrink in extent if blocked from shifting landward by the presence of developed areas [17, 18, 19].

In Florida, sustainable coastal habitats are critical drivers of both the economy and quality of life. The health and sustainability of coastal ecosystems control the future of the state’s recreational and commercial fisheries, recreational boating and diving, beach-related recreation, tourism, nature observation and other ecosystem dependent activities, collectively worth hundreds of billions of dollars a year to the state’s economy [20].

In many parts of the United States, human communities and infrastructure are increasingly vulnerable to storm surge effects in the face of SLR. Shepard *et al*. [21] found that even modest and probable SLR (0.5 m by 2080) vastly increases the numbers of people at risk (+47%) and property losses (+73%) from storm surge on the south shore of Long Island, New York. Intact coastal wetlands can cost-effectively help to buffer adjacent human communities from the impacts of storm surge [22, 23, 24, 25]. Human communities and coastal wetlands systems at several estuaries along the Florida Gulf of Mexico coast are especially vulnerable to SLR impacts due to their low-lying nature and the extensive development that blocks coastal wetlands from migrating to higher elevations. Improved information about the vulnerability of natural and human communities to SLR provides citizens and natural resource managers with the knowledge base necessary to take appropriate action and reduce consequences. Taking action now rather than waiting for impacts to accumulate can minimize hazards to human communities, disruptions to natural systems, and be more cost-effective in the long-term [26].

Among the tools developed to enhance our understanding of the effects of SLR on coastal ecosystems is the Sea Level Affecting Marshes Model (SLAMM; open source and available at: http://warrenpinnacle.com/prof/SLAMM/index.html). SLAMM was developed in the mid-1980s [27], with SLAMM 6.2 beta released in 2012. SLAMM employs a decision tree that incorporates geometric and qualitative relationships to simulate the dominant processes involved in changes to wetland systems and shoreline modifications during SLR. The five primary processes used to predict wetland fate with SLR are inundation, erosion, overwash, saturation and accretion. This model has been applied around the USA [28] with 50 individual applications from 20 states and 2 territories listed on the SLAMM-View website (http://www.slammview.org/). Several early applications used relatively low resolution (1.5 m contours) National Elevation Data (NED), which required SLAMM to extrapolate elevations based on land cover data such as provided by the National Wetlands Inventory (NWI). A recent analysis of SLAMM’s output uncertainty employed a generic evaluation framework and found that four model inputs including elevation, SLR historic trend, accretion rate and sedimentation rate controlled 88 to 91% of SLAMM 5’s output variance in predicting changes in the beach habitat at Eglin Air Force Base in Florida [29] and that model uncertainty could be reduced by using high resolution, LiDAR-derived elevation data and SLR historic trend information from NOAA tide stations with data collected over several decades. SLAMM has been shown to be a useful tool for simulating the effects of future SLR impacts on coastal wetland ecosystems. Geselbracht *et al*. [30] found that a hindcast of SLAMM closely matched changes observed over 20 years of field studies at Waccasassa Bay, Florida.

The study objective is to illustrate the utility of SLAMM-derived qualitative, quantitative and spatial information on potential changes to coastal ecosystems due to predicted SLR for informing adaptation planning. SLR impacts on coastal ecosystems were modeled at six estuarine study areas along the State of Florida’s Gulf of Mexico coast (3 SLR scenarios at each site through the year 2100: 0.7 m, 1.0 m and 2.0 m). In addition, uncertainty analyses were conducted on selected SLAMM input parameters at three study areas to better understand how these input parameters affect model output. Model predictions have been made available through workshops, reports, and an interactive, online decision support tool (coastalresilience.org). Study area stakeholders are using the newly generated information to develop and implement specific and locally–relevant adaptation strategies.

## Study Areas and Methods

### Study Areas

**Fig 1:**
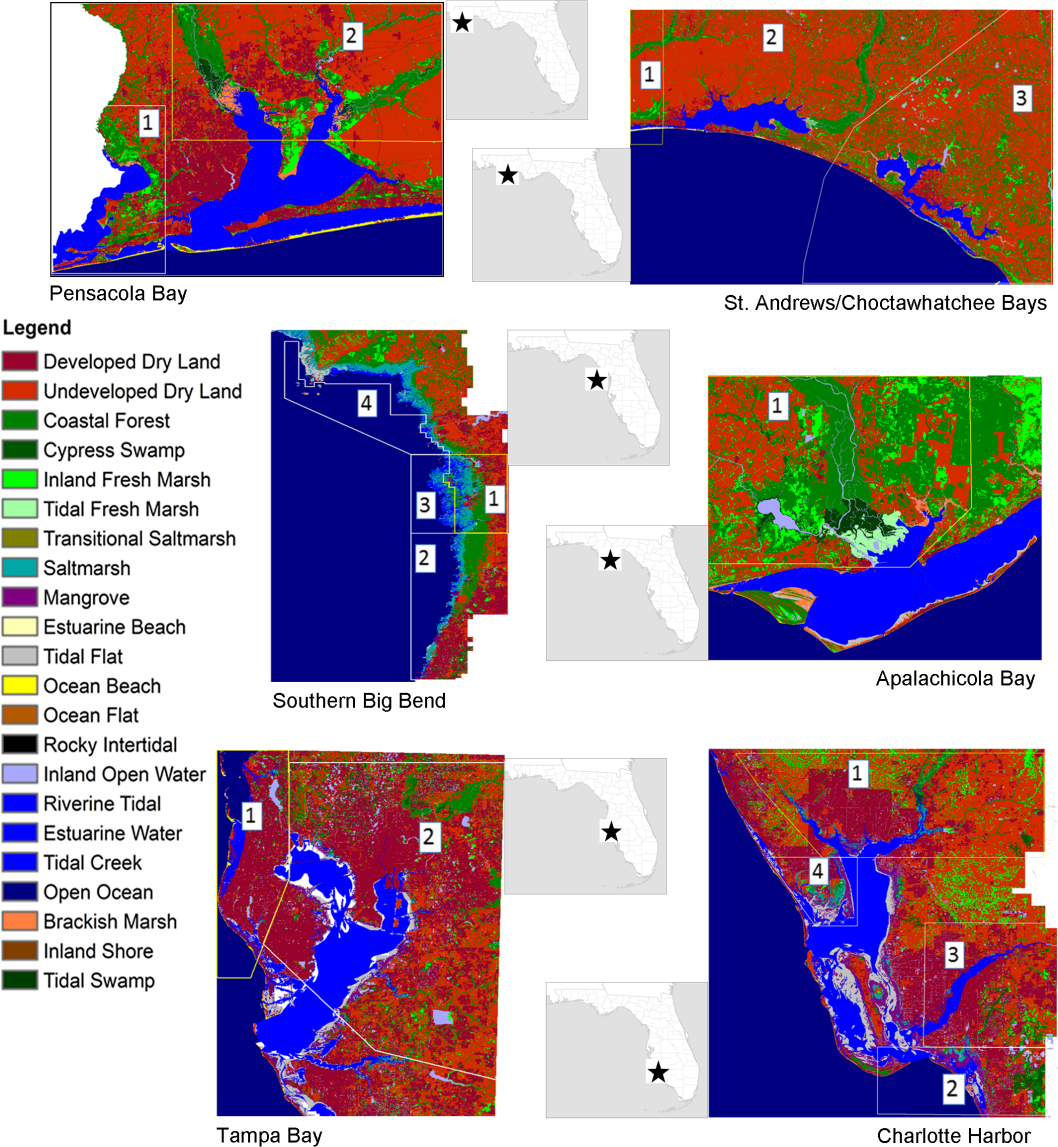
Project study areas, subsites and vicinity maps. The project study areas and vegetation raster inputs are illustrated in color for all study areas. The vegetation raster inputs represent the initial condition of coastal ecosystems used in the SLAMM analyses. Subsites are identified within study areas by numbers 1–4. Subsites were created where tidal parameters vary substantially from the global site (unmarked area) or where freshwater flow is substantial as along some river floodplains. Vicinity maps for each project study area along the Florida Gulf Coast are provided next to each study area map.

The analysis included six study areas that encompassed wetlands and uplands surrounding the major estuarine systems along the Florida Gulf of Mexico coast. Study areas were defined to encompass lands generally up to the 2 meter elevation contour and included three in the Florida Panhandle (Pensacola Bay, St. Andrews/ Choctawhatchee Bays and Apalachicola Bay), two in Southwest Florida ((Tampa Bay and Charlotte Harbor) and one along the transitional coast between the two regions (Southern Big Bend) (Fig 1). These estuaries were selected for focus because an earlier analysis had identified them as among the most biologically diverse and productive systems on Florida’s Gulf [31].

The more northerly study areas (Pensacola Bay to the Southern Big Bend) are similar in that a relatively higher percentage of their coastal wetlands are represented by coastal forest (18% to 44%) as compared to the more southerly study areas (Tampa Bay and Charlotte Harbor) with only 4% to 8% of their coastal wetlands represented by coastal forest (Fig 1; Table 1). The two most southerly study areas, Tampa Bay and Charlotte Harbor, are the only study areas with mangrove forest representing more than 1% of coastal wetlands. The Panhandle study areas receive relatively higher inflows from rivers than the other study areas and these rivers also carry relatively large amounts of inorganic sediments to the estuaries. The Southern Big Bend is unique among the study areas in that it receives most of its exceptionally large inflow from groundwater and it contains the majority of the saltmarsh represented by all of the study areas combined, approximately 77%.

**Table 1:** Study Area Characteristics. Some characteristics of the 6 study areas are presented including area, mean inflow, mean depth, average salinity, great diurnal tide, extent of coastal wetlands and the type and extent of the predominant subtidal habitat (not including bare sediments).

Study Area	Area (ha)	Mean Inflow (m^3^/s)	Mean Depth (m)	Average Salinity (ppt)	Great Diurnal Tide (m)	Coastal Wetlands (ha)	Predominant Subtidal Habitat excluding Bare Sediment (ha)
Pensacola Bay	351,679	328	4	23	0.4	> 63,000	seagrass; 1,800
St. Andrews/ Choctawhatchee Bays	1,581,932	241	5	25	0.16	238,353	seagrass; 6,500
Apalachicola Bay	274,525	824	3	22	0.7	> 117,000	oyster reef; > 1,400
Southern Big Bend	740,624	—-^a^	3^b^	15	0.6 to 1.2	> 90,960	seagrass; 153,470^c^
Tampa Bay	602,639	68	3	27	0.7 to 0.9	> 94,000	seagrass; 12,100
Charlotte Harbor	761,670	136	2	13	0.4	> 114,000	seagrass; > 23,900

^a^ Unknown.

^b^ Southern Big Bend is not an enclosed estuary. Open ocean areas adjacent to the coast are less than 3 m in depth.

^c^ This is likely an underestimate because seagrass has not been estimated in deeper waters due to insufficient water clarity.

Sources: [7, 32–44].

#### Pensacola Bay

**Fig 2:**
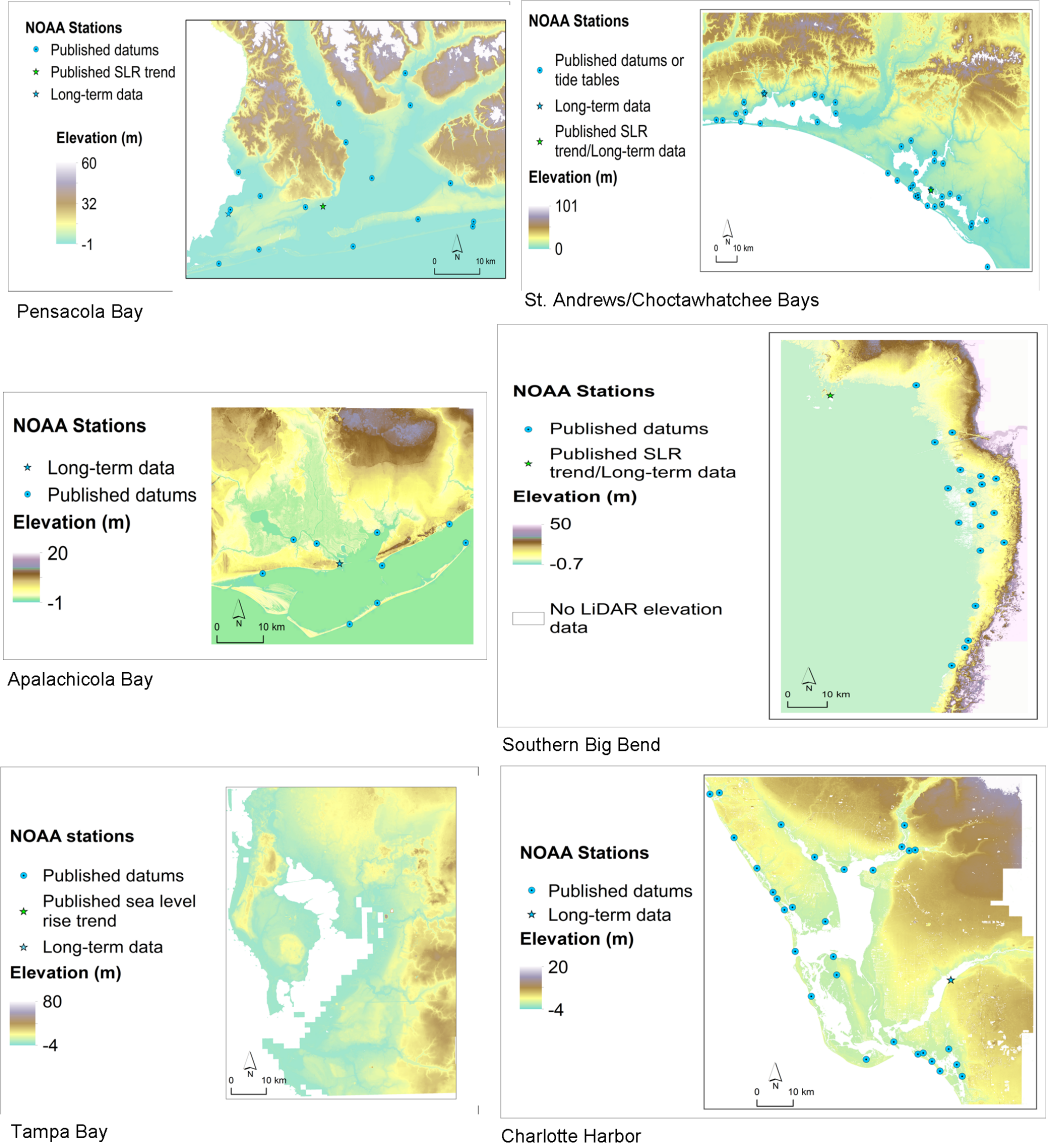
Digital Elevation Model (DEM) inputs and NOAA tide stations utilized for all study areas. Tidal parameters and location of stations informed the creation of subsites for each study area as illustrated in Fig 1.

Pensacola Bay is a relatively deep estuary along Florida’s Gulf Coast due to its location on the coast where the slope into the Gulf is steep and elevations close to shore are the highest among the study areas (Fig 2). Tidal fluctuations and flushing in Pensacola Bay are limited by a single narrow inlet opening into the Gulf of Mexico. Waters in the bay are stratified with the top fresh layer coming from river inflows, rainfall and sheet flow and a dense bottom, salty layer that extends up area rivers a few kilometers. Water mixing between the upper and lower reaches of the estuary is hampered by railroad and highway crossings [32]. The Florida portion of Perdido Bay, which is fed by the Perdido River, is also part of this study area.

#### St. Andrews/Choctawhatchee Bays

The St. Andrews/Choctawhatchee Bays study area combines two adjacent bay systems (Fig 2). The Choctawhatchee River, the main source of freshwater flowing into Choctawhatchee Bay, has a broad floodplain and is subject to seasonal flooding and heavy sediment loads. In addition, the bay is fed by the Floridan aquifer, as well as blackwater and spring-fed tributaries. No major rivers flow into St. Andrews Bay, however it does receive freshwater flow from a number of other sources including creeks and bayous. Gulf of Mexico water dominates the bay due to the restricted amount of freshwater inputs [32].

#### Apalachicola Bay

A key feature of Apalachicola Bay system is the large fluvial delta formed by the Apalachicola River, the largest freshwater source flowing into the Bay and the largest river by flow in Florida. Sediment has accumulated in this delta system and into the bay since the Holocene from a large drainage basin that includes much of Georgia and Alabama [33]. The bay is shallow and fronted by a nearly continuous barrier island system. Due to the large flows of the Apalachicola River, the bay experiences long periods of low salinity [32], making it an ideal environment for oyster reefs to thrive.^.^

#### Southern Big Bend

The Southern Big Bend region is not a typically shaped estuary (Fig 2). It is low relief coastline that functions as an estuary due to the extensive freshwater sheet flow that enters the Gulf of Mexico in this area. Surface water salinity approximately 4 km offshore averages about 15 ppt [32]. Only small, short, spring fed rivers flow into the Gulf in this region. Subtidal habitats in the region include oyster reef and a portion of one of the world’s largest continuous seagrass beds.

#### Tampa Bay

Tampa Bay is a shallow estuary located in a transition zone between warm-temperate and tropical biogeographic provinces. As a consequence of its location, the bay supports a highly diverse flora and fauna [33]. The bay is fed by streams that carry little or no sediment [32]. Springs and underlying aquifers also provide groundwater to the bay. The northern portion of the bay is highly urbanized whereas much of the southern portion remains in a more natural state.

#### Charlotte Harbor

Charlotte Harbor is a shallow, subtropical estuary made up of a number of bays and surrounded by large areas of protected coastal ecosystems. The estuary is the second largest in Florida and is separated from the Gulf of Mexico by barrier islands. Two major inlets and several smaller passes connect the estuary to the Gulf. The shoreline is mostly undisturbed except along the Caloosahatchee River where urban and residential development has proliferated [35].

## Methods

### Simulation Modeling of SLR impacts on Coastal Ecosystems

SLAMM was applied to each study area using three SLR scenarios through the year 2100, the IPCC A1B maximum scenario (0.7m), and two additional scenarios, 1 m and 2 m to capture a range of scientifically supported scenarios. Global SLR scenarios are appropriate to use in Florida because local SLR has generally mimicked global mean SLR over the last couple of decades with some variation along Florida’s Gulf Coast (1.6 to 3.19 mm per year) as calculated from tide gauge records [7]. The IPCC A1B maximum scenario is based on a mid-range of climate scenarios and takes into account thermal expansion of the ocean and the melting of glaciers, but includes little contribution due to the melting of the Greenland and Antarctic ice sheets [1]. More recent semi-empirical modeling approaches have found that the IPCC scenarios are likely underestimates. Rahmstorf *et al*. [5] found that sea level has been rising 80% faster over the last several decades than IPCC projections (3.4 mm/yr vs. 1.9 mm/yr) as indicated by satellite imagery. Vermeer and Rahmstorf [6] demonstrated that the global mean sea level is linked to global temperature and projected that sea level would rise as much as three times faster than IPCC 2100 projections. Grinsted *et al*. [3] projected sea level would rise by 0.9 to 1.3 m by 2100 using a simple model forced by temperatures calibrated by using an inverse Monte Carlo method. Pfeffer *et al*. [43] employed climate modeling constrained by glaciological conditions and found sea level could potentially rise from 0.8 m to 2.0 m by 2100 with 0.8 m being the most plausible scenario and 2.0 m being the upper, still feasible, limit. In addition, two protection scenarios were run to provide insights into how coastal ecosystems might respond in more and less developed states: protect developed dry land and developed dry land allowed to transition (i.e., unprotected). SLAMM requires that each study area be divided into cells of equal area. Each cell is associated with an elevation, slope, aspect and initial wetland (e.g., vegetation) type based on 23 of the National Wetlands Inventory (NWI) categories. SLAMM employs a decision tree that incorporates geometric and qualitative relationships to simulate the dominant processes involved in changes to wetland systems and shoreline modifications during SLR. The five primary processes used to predict wetland fate with SLR are inundation, erosion, overwash, saturation and accretion. Depending on how SLR affects the cell and adjacent cells over the study period, the cell may convert to another wetland type. A complete description of the model and implementation information can be found in the model technical documentation [44] and user guide [45]. Variations from the general methods described below are detailed in the site specific subsections that follow. Esri ArcGIS software, ArcInfo versions 10 or 10.1, was used to do all geoprocessing needed to prepare raster inputs. Study area boundaries were established to encompass at least 2 meters of elevation, and in some cases to integrate with other projects.

#### Model Inputs

SLAMM requires two types of inputs to simulate changes in coastal wetlands due to SLR: raster (i.e., spatial) data and numeric site parameters. The raster datasets are uploaded to SLAMM via the user interface. The numeric site parameters are entered into the SLAMM user interface in a tabular format. Together the raster data and numeric parameters describe the site to be modeled. In addition to the two types of inputs, the user specifies SLR scenarios and selects optional model switches that control various aspects of the simulation via the user interface.

SLAMM requires three raster data inputs, vegetation, elevation and slope. The source of these inputs and any required processing are described below:

The comprehensive, statewide vegetation dataset, the Cooperative Land Cover Map v1.1 (CLC) available from the Florida Natural Areas Inventory (FNAI) in vector format) [46] was used to create the vegetation input raster. The vegetation input raster defines the coastal ecosystems in the study area to be analyzed and includes wetland and dry land categories. The CLC vegetation categories were translated into the SLAMM required vegetation categories using a table provided in the SLAMM technical documentation [44]. The modified crosswalk table between the CLC and SLAMM vegetation categories is provided in S1 Table. The CLC data do not include tidally influenced categories (e.g., brackish marsh and tidal swamp). Tidally influenced areas were identified using the most recent NWI data. All crosswalks, edits or attribute modifications were done to the vector features. Once all changes were made to the vegetation categories, the data was converted to a raster that aligned with the Digital Elevation Model (DEM). Throughout the document the more commonly recognized wetland ecosystem names are used (as opposed to the SLAMM category names) as follows: saltmarsh in place of regularly flooded marsh, brackish marsh in place of irregularly flooded marsh, coastal forest in place of swamp, and transitional saltmarsh in place of scrub shrub. Maps of the initial vegetation raster for each study area are presented in Fig 1.

High resolution, LiDAR-derived Digital Elevation Models (DEMs) available from a water management district or the Florida Division of Emergency Management were used to create the required elevation raster for each study area (www.floridadisaster.org/gis/lidar/). A summary of the source data used for each study area is provided in S2 Table. For each study area, DEMs were mosaicked together (if necessary), resampled to either 15 or 30 m cell size, re-projected to the site’s coordinate system and clipped to the study area boundary. Any raster cells with a NoData value (i.e., no elevation data recorded) that fell under a SLAMM open water category were set to zero, so that the model’s decision tree would not operate on those cells. Maps of the elevation rasters for each study area are presented in Fig 2.

The third required raster input, a slope raster, was created from the final DEM using the Slope tool in the Spatial Analyst extension of ArcGIS.

Each study area was modeled using the best available site-specific numeric parameters. Twenty-one SLAMM required numeric input parameters were defined for each study area and subsite and included the dates the elevation and vegetation data were collected and site specific tidal, SLR, erosion, accretion, sedimentation and overwash parameters (S3 Table). Where numeric inputs varied spatially across a study area or where large freshwater flow areas were present subsites were created to take these differences into account (Fig 1).

SLAMM allows for each subsite to have its own set of numeric parameters, thus allowing for spatial variation to be modeled. In addition to subsites defined due to variations in numeric input parameters, freshwater flow sites were defined in some study areas.

### Running the model

For each study area, the three SLR scenarios (0.7 m, 1 m and 2 m) were run with 25 year time steps through the year 2100. GIS output was obtained for each time step with every scenario. Each scenario was run twice, once with developed and undeveloped dry land allowed to become inundated, and once with developed dry land protected from inundation at all study areas except the St. Andrews/Choctawhatchee Bay study area. The latter scenario simulated protecting existing development (e.g., through shoreline hardening), a likely SLR adaptation response. All scenarios were run using the optional connectivity algorithm. This option allows dry land and freshwater wetlands to become inundated with saltwater only if there is a connection to a saltwater source.

### Uncertainty Analysis

The numerical parameters for each study area’s SLAMM simulation were developed with data available at the time. For some sites, site specific parameter information was available for all numerical input parameters (e.g., saltmarsh accretion rate, beach sedimentation rate, etc.). For other sites, site specific parameter information was not available or only available for some of the numerical input parameters. The simulated results of the scenarios presented below have all of the types of uncertainty inherent to simulation models [47]. To address the question of input uncertainty, an uncertainty analysis module was added to version 6 of SLAMM [44].

SLAMM’s uncertainty analysis module was used to examine input uncertainty for selected parameters at three study areas. The uncertainty analysis module allows users to specify a distribution for any input parameter, or multiple parameters, and performs a Monte-Carlo analysis. The number of iterations was specified using a non-random seed. A limitation of SLAMM's uncertainty module is that the values drawn from the distribution are multipliers applied to a single value of the parameter in question. That is, the parameter value to which the multiplier is applied does not change even if that parameter changes across subsites.

The uncertainty analyses were limited by computer resources. With small cell sizes of 15 or 30 meters and available computer capacity, 100 iterations could take 24 hours or more. As a result, the effects of input uncertainty on 2 or 3 numeric parameters were examined at the study areas evaluated (Pensacola Bay, Southern Big Bend and Tampa Bay). The number of iterations was set to 100 for the 1 m SLR scenario. While 100 iterations can reveal trends, this number of iterations may not be enough to capture extremes. For the Pensacola Bay study area, the uncertainty module was run with a representative subset of the study area to shorten the runtime of the 100 iterations. For Tampa Bay study area, three parameter distributions were included in the 100 model iterations. All uncertainty analyses were run on the 1 m SLR scenario with developed dry land allowed to transition.

Three parameters for uncertainty that appeared influential in simulation outcomes were selected for the uncertainty analyses: marsh accretion, sedimentation and salt elevation. Generally, distributions used were plus or minus a likely spread from the input value based on values from the literature at nearby sites. Triangular distributions were used when there was a basis that a value for a parameter was more likely. In some cases, minimum and maximum values were chosen so that the distribution would encompass values from research that indicated a wider range as noted in Table 2.

**Table 2:** Parameters and their statistical distribution input into the SLAMM uncertainty analysis module. U = Uniform distribution (minimum, maximum); T = Triangular distribution (minimum, most likely, maximum).

Study Area	SLAMM Parameter	Input Value	Distribution and Source
Pensacola	Saltmarsh Accretion (mm/yr)	2.25	T(0.9, 3.2, 8) [29]
Pensacola	Brackish marsh Accretion (mm/yr)	3.75	U(3,4) [29]
Southern Big Bend	Saltmarsh Accretion (mm/yr)	7.2	T(0.7, 7.2, 7.6) [48]
Southern Big Bend	Beach Sedimentation Rate (mm/yr)	0.5	U(0.375, 0.625)
Tampa Bay	Salt Elevation (m above MTL)	0.55	U (0.495, 0.605)
Tampa Bay	Saltmarsh Accretion (mm/yr)	2.25	T(0.9, 2.25, 8.0) [29]
Tampa Bay	Beach Sedimentation Rate (mm/yr)	2.7	T (0.01, 2.5, 5) [29]

## Results

### Simulation Modeling of SLR impacts on Coastal Ecosystems

To simplify reporting of the results, focus was placed on the 1 m SLR scenario, which is in the mid-range of what are considered plausible scenarios [43]. Unless otherwise noted, the scenarios discussed in this section are with developed dry land protected from transitioning to another habitat type. Results of additional model runs (i.e., developed dry land allowed to transition) are summarized and provided in supporting information as noted below. At all the study areas, SLAMM predicts modest to substantial changes in coastal ecosystems due to SLR.

### Pensacola Bay Study Area

**Fig 3:**
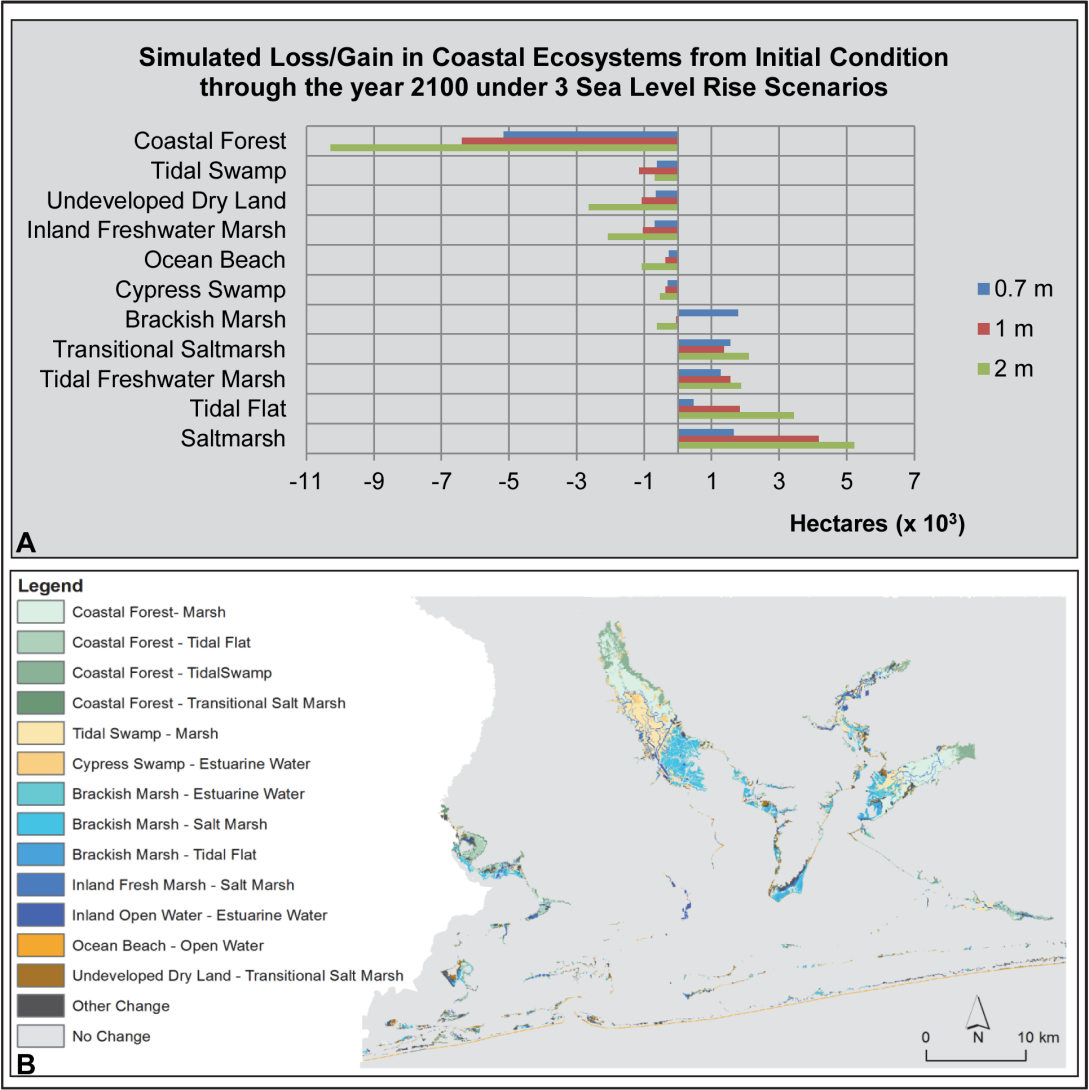
Pensacola Bay Area SLAMM Results. (A) Bar graph of loss/gain of coastal ecosystems under 3 sea level rise scenarios. (B) Map of SLAMM results illustrating the change in coastal ecosystem types (from/to) under a 1 m sea level rise scenario.

In the Pensacola Bay study area (including the Florida portion of Perdido Bay), SLAMM forecast simulations estimated that 18% (-6,408 ha) of study area coastal forest would be lost under the 1 m SLR scenario (Table 3). This represents the largest predicted spatial loss of a coastal ecosystem in the Pensacola Bay study area under this SLR scenario. Most of this predicted coastal forest loss occurred in the lower floodplains of the rivers emptying into Pensacola and Perdido bays (the Escambia, Yellow, Blackwater and Perdido rivers; Fig 3). Other types of forested wetlands predicted to be lost under 1 meter of SLR included tidal swamp and cypress swamp with losses of 1,157 ha (-44%) and 370 ha (-28%), respectively. Much of the brackish marsh that is currently present in the river delta areas was predicted to transition to estuarine waters, tidal flats and saltmarsh. However, only a net loss of 58 ha of brackish marsh was reported by SLAMM as this ecosystem type was predicted to replace much of the forested wetlands lost with 1 m of SLR. Other coastal systems predicted to experience loss under the 1 m scenario included ocean beach (-17%; -376 ha), inland freshwater marsh (-11%; -1,048 ha) and undeveloped dry land (1%; -1,075 ha). Conversely, some coastal ecosystems were predicted to increase in size (Table 3; Fig 3) including saltmarsh (+4,166 ha), tidal flat (+1,826 ha), tidal freshwater marsh (+1,536 ha) and transitional saltmarsh (+1,357 ha). Percentage increases predicted in these systems are not presented here as they are not meaningful due to the relatively small spatial extent of these systems under current conditions. Overall, SLAMM predicted a modest net loss of study area coastal wetland ecosystems (-512 ha; <-1%).

**Table 3:** Quantitative SLAMM Results–coastal ecosystem change under a 1 meter SLR scenario through the year 2100, developed dry land protected from changing.

	Pensacola Bay		St. Andrews/ Choctawhatchee Bays		Apalachicola Bay		Southern Big Bend		Tampa Bay^1^		Charlotte Harbor	
Coastal Ecosystem	Change (ha)	% Change	Change (ha)	% Change	Change (ha)	% Change	Change (ha)	% Change	Change (ha)	% Change	Change (ha)	% Change
**Undeveloped Dry Land**	-1,075	-1%	-4,360	-0.6%	-3,084	-5%	-9,066	-14%	-3,868	-4%	-6,724	-4%
**Coastal Forest**	-6,408	-18%	-10,241	-6%	-20,857	-27%	-24,655	-49%	-2,413	-8%	-4,336	-24%
**Tidal Flat**	1,826	2021%	3,747	312%	3,020	156%	1,333	34%	-14,627	-93%	-20,524	-96%
**Inland Freshwater Marsh**	-1,048	-11%	-1,125	-3%	-4,379	-20%	-758	-10%	-276	-2%	-446	-1%
**Tidal Swamp**	-1,157	-44%	na	na	-3,357	-56%	-353	-80%	na	na	-376	-96%
**Cypress Swamp**	-370	-28%	-123	-1%	-644	-24%	-140	-9%	-36	-0.4%	-29	-0.3%
**Ocean Beach**	-376	-17%	50	6%	-94	-26%	40	217%	-130	-12%	-201	-27%
**Estuarine Beach**	17	na	-595	-33%	165	744%	57	5702%	-25	-13%	38	na
**Tidal Freshwater Marsh**	1,536	Na	-188	-41%	2,303	60%	-12	-37%	-41	-94%	na	na
**Brackish Marsh**	-58	-2%	-1,809	-41%	7,834	269%	107	10668%	-180	-92.3%	**na**	**na**
**Mangrove Forest**	na	na	na	na	na	na	-121	-44%	4,453	63%	8,251	34%
**Transitional Saltmarsh**	1,357	na	6,547	na	3,081	na	11,592	na	-14	-17%	495	na
**Saltmarsh**	4,166	na	4,862	na	10,503	na	15,736	50%	-1,491	-73%	-342	-5%
**Net Change in Wetlands:**	-512	-<1%	+1,125	+0.5%	-2,425	-2%	2,826	3%	-14,780	-19%	-17,507	-15%

^1^Results exclude tidal flats with no elevation data (approximately 10,000 ha).

Under the SLAMM scenarios with developed dry land allowed to transition (S4 Table), SLAMM simulated losses of developed dry land at 1%, 2%, and 5% with the 0.7 m, 1 m and 2 m scenarios, respectively. The direction and magnitude of change simulated for coastal wetlands under the 1 m SLR scenario were similar to the developed dry land protected scenarios with the exception that simulated gains of transitional saltmarsh, saltmarsh and tidal flat were larger and simulated losses tidal freshwater marsh and ocean beach were smaller.

### St. Andrews/Choctawhatchee Bays

**Fig 4:**
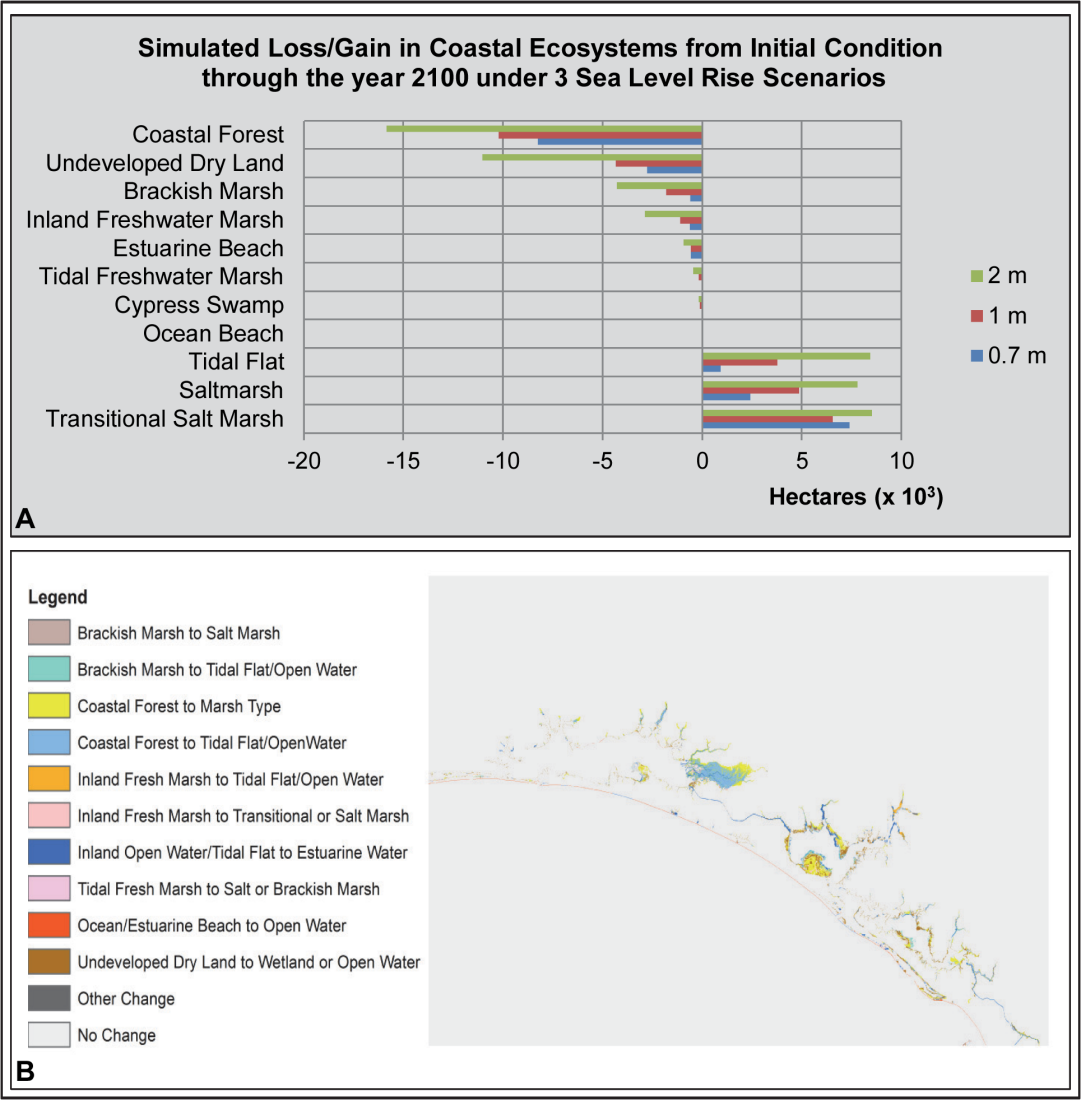
St. Andrews/Choctawhatchee Bays Study Area SLAMM Results. (A) Bar graph of loss/gain of coastal ecosystems under 3 sea level rise scenarios. (B) Map of SLAMM results illustrating the change in coastal ecosystem types (from/to) under a 1 m sea level rise scenario.

SLAMM forecast simulations for St. Andrews/Choctawhatchee Bays study area estimated that approximately 6% of study area coastal forest was lost under the 1 m SLR scenario (Table 3). However, this represents over 10,000 ha of coastal forest predicted to be lost to this system. Most of this estimated loss would take place in the lower Choctawhatchee floodplain and the southern shore of the West Bay portion of St. Andrews Bay (Fig 4). The coastal forest loss in these areas was predicted to transition to various marsh types, tidal flat or open water. Approximately, 4,360 ha (-0.6%) of undeveloped dry land was predicted to transition to various wetland types or open water. Much of this change was predicted to take place along the St. Andrews Bay shoreline. Other coastal wetland losses exceeding 1,000 ha predicted by SLAMM in this study area included brackish marsh (-1,809 ha; -41%) and inland freshwater marsh (-1,125 ha; -3%). Conversely, SLAMM predicted substantial gains in extent for transitional saltmarsh (+6,547 ha; percent not available), saltmarsh (+4,862 ha; percent not available) and tidal flat (+3,747 ha; +312%). Ocean beach was also predicted to increase in area, but to a much lesser extent (+50 ha; +6%). Overall, SLAMM predicted a modest net gain of study area coastal wetlands under a 1 m SLR, +1,125 ha (+0.5%).

### Apalachicola Bay Study Area

**Fig 5:**
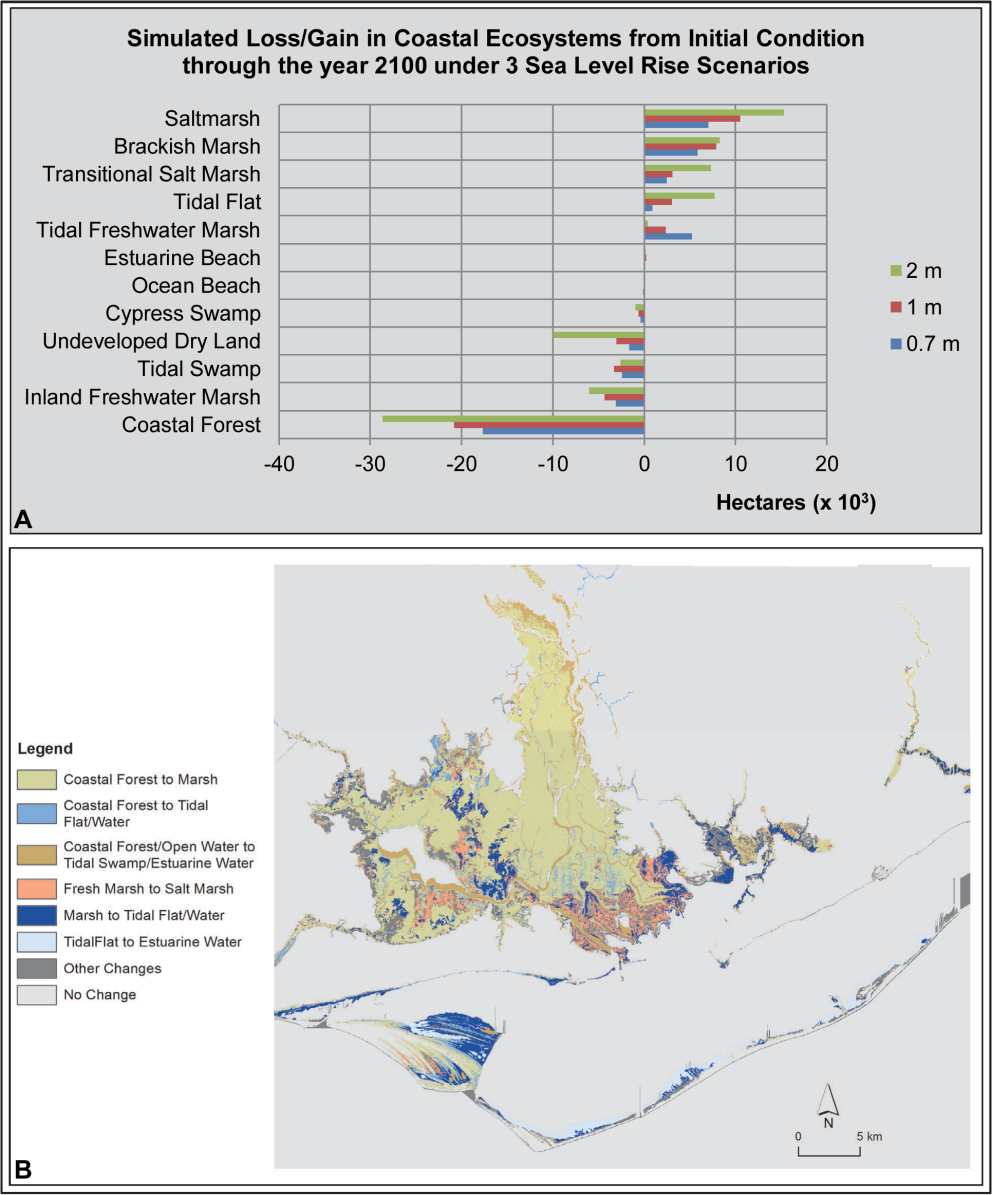
Apalachicola Bay Area SLAMM Results. (A) Bar graph of loss/gain of coastal ecosystems under 3 sea level rise scenarios. (B) Map of SLAMM results illustrating the change in coastal ecosystem types (from/to) under a 1 m sea level rise scenario.

As with the previous two study areas discussed, the Apalachicola Bay study area was predicted by SLAMM to lose a large amount of coastal forest under 1 m of SLR (-20,857 ha or -27%; Table 3). The loss is much larger in this study area is likely because the Apalachicola River is much larger than the rivers feeding into the estuaries of the other study areas and has a much larger lower floodplain. The majority of the coastal forest lost was predicted to transition into various marsh types (Fig 5). Other coastal ecosystems predicted by SLAMM to experience large percent losses of spatial extent included tidal swamp (-56%; -3,357 ha), ocean beach (-26%; -94 ha) and inland freshwater marsh (-20%; -4,379 ha). Undeveloped dry land was predicted by SLAMM to lose 3,084 ha (- 5%) of this ecosystem type in the study area.

The loss of coastal forest to marsh noted above resulted in large predicted increases in saltmarsh (+10,503 ha; percent not available), brackish marsh (+7,834 ha; +269%) and transitional saltmarsh (+3,081 ha; percent not available) and tidal freshwater marsh (+2,303 ha; +60%; Table 3). In particularly low-lying areas of the study area, currently existing marsh was predicted to transition to tidal flat resulting for a net gain of this ecosystem type (+3,020 ha; 156%; Fig 5). SLAMM predicted a net loss of study area coastal wetlands under a 1 m SLR for this study area (-2,425 ha; -2%).

A comparison of the scenarios run with developed dry land allowed to transition revealed few differences with the developed land protected scenarios (S5 Table) except with the developed dry land category. Developed dry land was lost in all 3 unprotected scenarios with approximately, 5%, 9% and 25% lost under the 0.7 m, 1 m and 2 m scenarios, respectively. Estuarine beach was the only coastal wetland ecosystem with notable difference between the developed dry land allowed to transition and the developed dry land protected scenarios. Under the allowed to transition scenario, substantially more estuarine beach was gained, 165 ha (+939%) as compared to the developed dry land protected scenario, 208 ha (+744%) for 1 m scenario.

### Southern Big Bend Study Area

**Fig 6:**
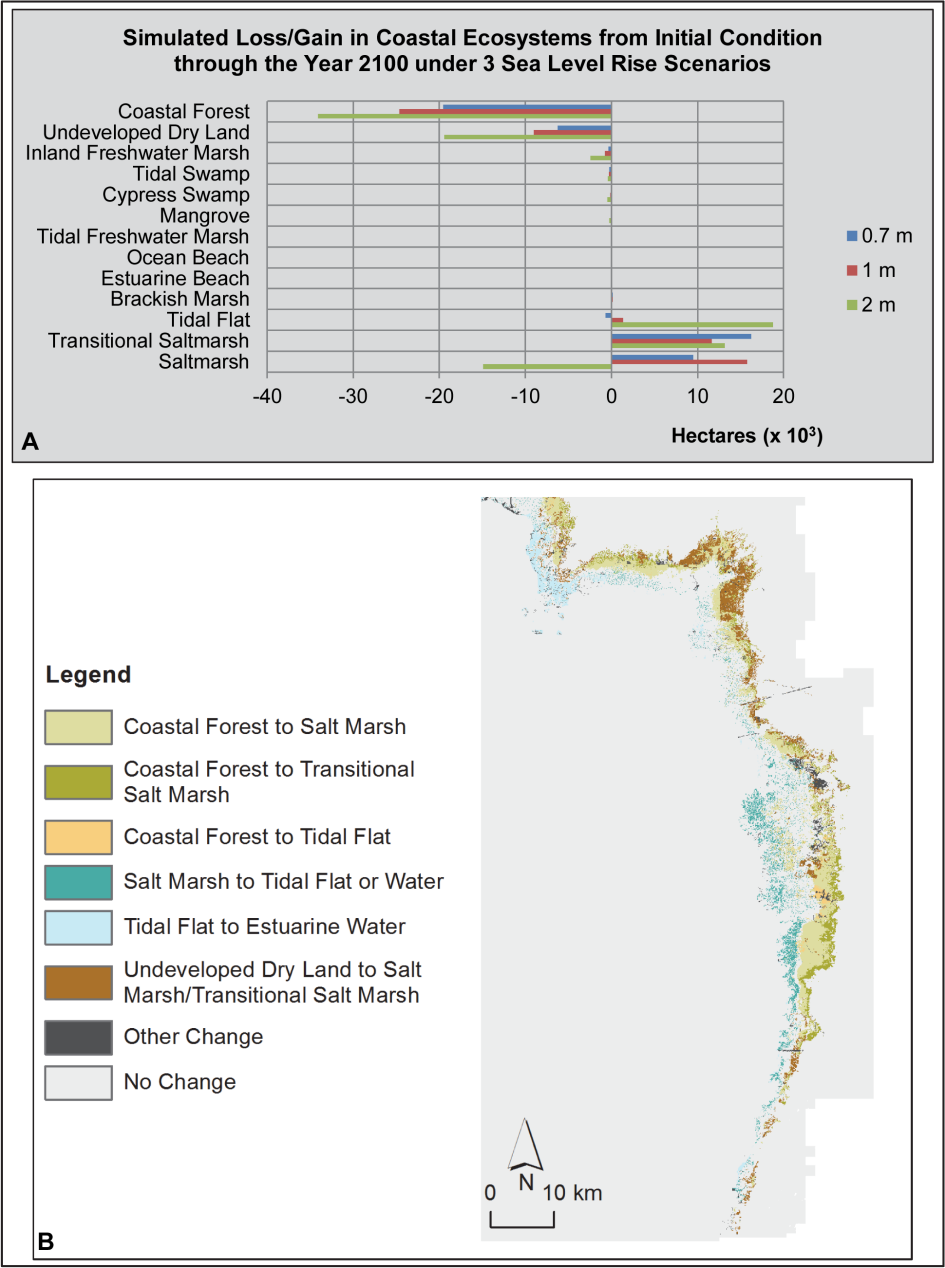
Southern Big Bend Study Area SLAMM Results. (A) Bar graph of loss/gain of coastal ecosystems under 3 sea level rise scenarios. (B) Map of SLAMM results illustrating the change in coastal ecosystem types (from/to) under a 1 m sea level rise scenario.

Similar to the Florida Panhandle study areas, SLAMM predicted a large loss of coastal forest extent in the Southern Big Bend study area (-24,655 ha; -49%; Table 3). A large loss in extent was also predicted for undeveloped dry land (- 9,066 ha; -14%). This loss in extent of undeveloped dry land with 1 m of SLR is the largest percent loss of all six study areas and is a result of the extensive low elevation lands in this study area. Most of the undeveloped dry land was lost in the vicinity of Waccasassa Bay and was predicted by SLAMM to transition to saltmarsh and transitional marsh (Fig 6). Inland freshwater marsh was predicted by SLAMM to lose a moderate amount of spatial extent (-758 ha; -10%).

SLAMM predicted an increase in saltmarsh (+15,732 ha; +50%), transitional saltmarsh (+11,592 ha) and tidal flat (+1,333 ha; +34%). Percentage changes are not reported for some coastal habitats due to their very low representation currently in the study area (approximately 1 ha or less) making their percent gain information less meaningful. A net gain of study area coastal wetlands was predicted by SLAMM in the Southern Big Bend study area (+2,826 ha; +3%) and is primarily a consequence of coastal forest loss.

As with the St Andrews/Choctawhatchee Bays study area, there appeared to be a threshold reached somewhere between 1 m and 2 m of SLR by 2100 at this study area (Fig 6). While SLAMM predicted a substantial gain of saltmarsh under the 1 m SLR scenario as noted above, under the 2 m SLR scenario saltmarsh was predicted to lose 14,933 ha. Conversely, tidal flat was predicted to experience a substantial gain in spatial extent under the 2 m SLR scenario, +18,710 ha. The loss of undeveloped dry land more than doubled under the 2 m SLR scenario as compared to the 1 m SLR scenario (loss of 19,465 ha versus 9,066 ha).

Under the SLAMM scenarios with developed dry land allowed to transition (S5 Table), SLAMM simulated loss of developed dry land at 5%, 7%, and 16% with the 0.7 m, 1 m and 2 m scenarios, respectively. For wetland systems, the direction and magnitude of change simulated were similar to the developed dry land protected scenarios with the following notable exceptions: there were larger simulated gains in ocean beach (+200 ha vs. +40 ha) and estuarine beach (+222 ha vs. +57 ha) under the 1 m SLR scenario with developed dry land allowed to transition versus developed dry land protected from change.

### Tampa Bay Study Area

**Fig 7:**
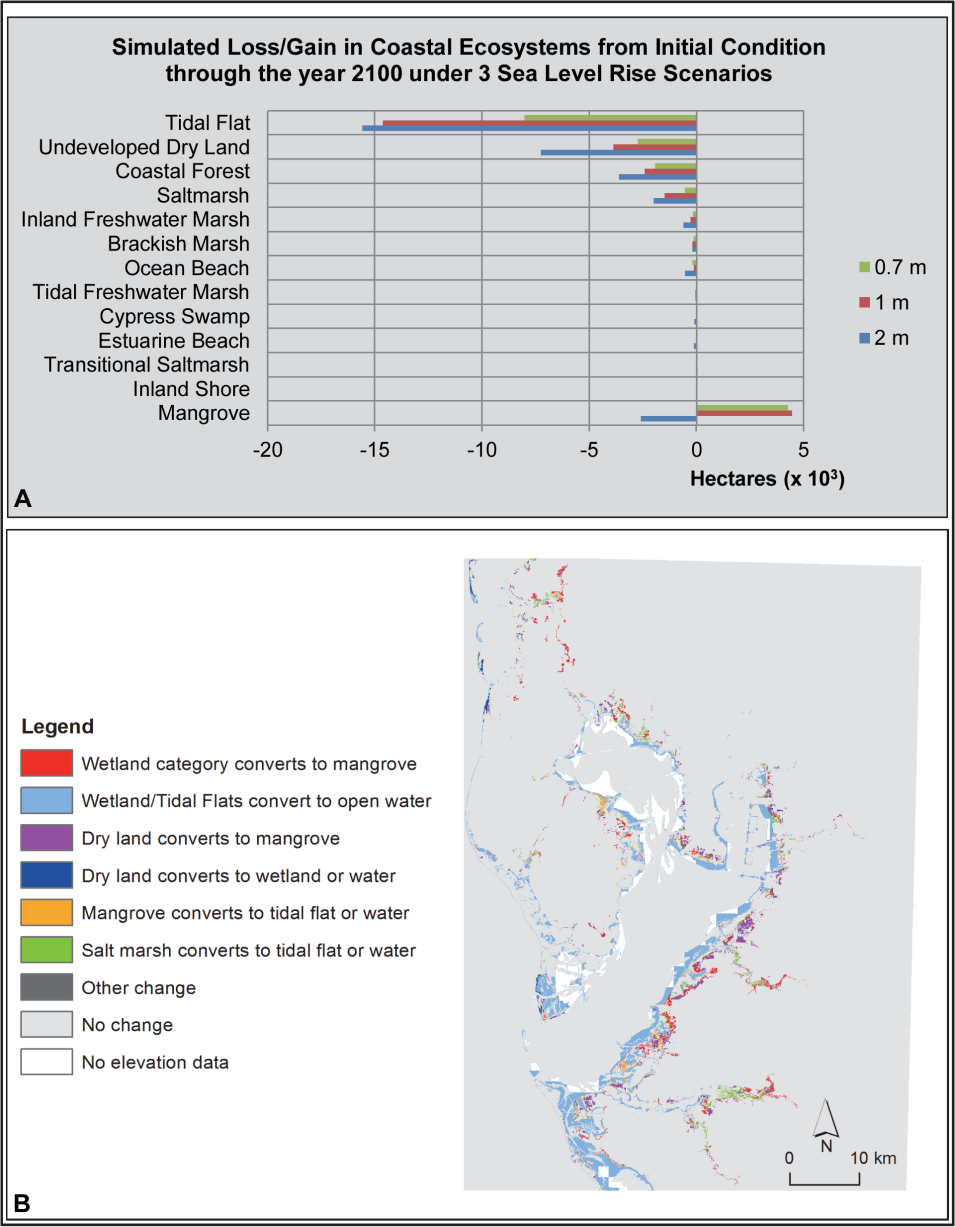
Tampa Bay Study Area SLAMM Results. (A) Bar graph of loss/gain of coastal ecosystems under 3 sea level rise scenarios. (B) Map of SLAMM results illustrating the change in coastal ecosystem types (from/to) under a 1 m sea level rise scenario.

SLAMM predicted modest to substantial changes in coastal ecosystems due to SLR in the Tampa Bay study area under a 1 m SLR scenario (Table 3). The most notable change was the transition of tidal flat to open water (i.e., shallow subtidal habitat; -14,627 ha; -93%; Fig 7). This predicted loss of tidal flat in this sub-tropical bay system was comparable in magnitude to the predicted loss of coastal forest in the more temperate study areas to the north. This loss of tidal flat is an underestimate as elevation data was not available for 10,290 ha of tidal flats in the study area. Table 3 values for tidal flats are adjusted so that they do not include these areas, which remained unchanged in the simulation; however, it is realistic to assume these areas would also be inundated. Other predicted losses in coastal ecosystems under the 1 m SLR scenario included undeveloped dry land (-3,867 ha; -4%), coastal forest (-2,413 ha; 8%) and a large portion of the modest amount of saltmarsh remaining in this system (-1,490 ha; -73%). Several other coastal wetland ecosystems were also predicted by SLAMM to lose spatial extent, but all by less than 300 ha under the 1 m SLR scenario: inland freshwater marsh, brackish marsh, ocean beach, tidal freshwater marsh, cypress swamp, estuarine beach and transitional saltmarsh. Total net loss of study area coastal wetlands was predicted to be 14,783 ha or 19%. This is a large net loss of coastal wetlands in the study area compared to the more temperate study areas covered above. Mangrove forest was the only coastal wetland ecosystem predicted by SLAMM to increase in spatial extent in this scenario (Table 3).

As with other study areas, there appeared to be a threshold between 1 m and 2 m of SLR by 2100: with 1 m SLR, mangrove forest gained 4,453 ha or 63% in extent. However, at the rate of 2 m SLR by 2100, mangrove forest was predicted by SLAMM to lose 2,589 ha in extent or 37%. In addition, loss of undeveloped dry land nearly doubled from the 1 m SLR scenario to the 2 m SLR scenario (-3868 ha versus -7263 ha). Under the 1 m scenario, several of the lowest elevation wetland types transitioned into tidal flat. Coastal forest and undeveloped dry land transitioned to mangrove forest and some mangrove forest was lost to shallow subtidal habitat.

Under the SLAMM scenarios with developed dry land allowed to transition (S5 Table), approximately 2%, 4%, and 11% of developed dry land was predicted to be lost under the 0.7 m, 1 m and 2 m scenarios, respectively. The direction and magnitude of change simulated for coastal wetlands was similar to the developed dry land protected scenarios with the exception that under the 1 m SLR scenario simulated changes in mangrove forest were much larger (+11,912 versus +4,452 ha).

### Charlotte Harbor Study Area

**Fig 8:**
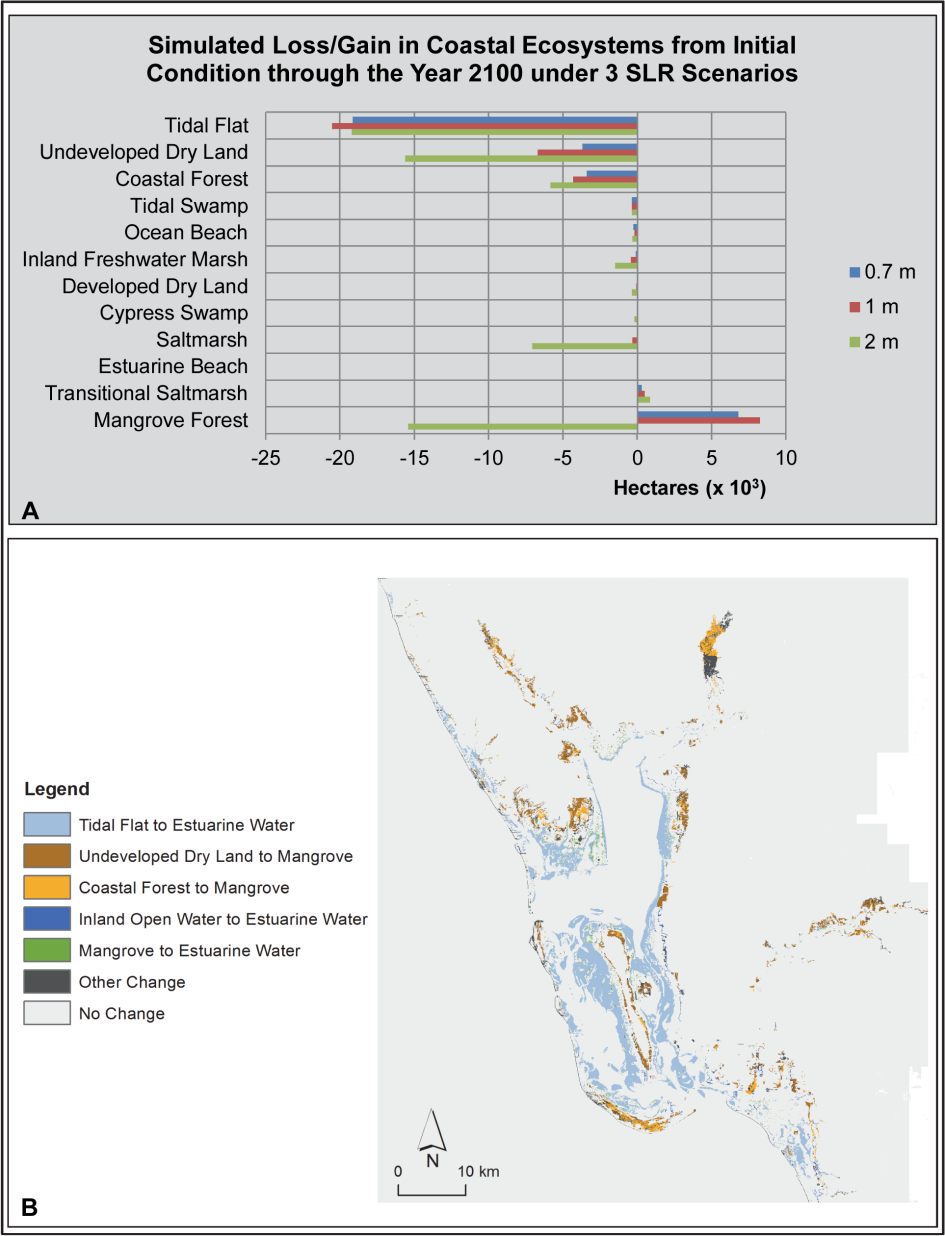
Charlotte Harbor Study Area SLAMM Results. (A) Bar graph of loss/gain of coastal ecosystems under 3 sea level rise scenarios. (B) Map of SLAMM results illustrating the change in coastal ecosystem types (from/to) under a 1 m sea level rise scenario.

As with the Tampa Bay study area, sub-tropical Charlotte Harbor was predicted by SLAMM to lose the majority of the tidal flat ecosystem now present in this system under a 1 m SLR scenario (-29,524 ha; -96%; Table 3). These tidal flats were predicted to transition into open water (i.e., shallow subtidal habitat; Fig 8). Losses of coastal ecosystems in excess of 1,000 ha predicted by SLAMM under a 1m SLR scenario included coastal forest (-4,336 ha, -24%) and undeveloped dry land (-6,724 ha; -4%). SLAMM predicted that the coastal forest and undeveloped dry land lost primarily transitioned into mangrove forest (Fig 8). Other coastal wetland ecosystems predicted by SLAMM to lose spatial extent were estimated to lose less than 450 ha (inland freshwater marsh, tidal swamp, saltmarsh, ocean beach and cypress swamp). Although the loss of these wetland ecosystems is relatively small in area, in some cases these losses represented a large percentage of that system in the study area, e.g., tidal swamp, -96% and ocean beach, -27%). Increases in spatial extent under the 1 m SLR scenario were predicted by SLAMM for mangrove forest (+8,251 ha; +34%), transitional saltmarsh (+495 ha) and estuarine beach (+38 ha; Table 3). It was not possible to calculate a percent increase for transitional saltmarsh and estuarine beach as these ecosystem types were not present in the initial condition. As with the sub-tropical Tampa Bay study area, SLAMM predicted a large net loss of coastal wetlands (-17,507 ha; -15.3%) in this study area as compared to the more temperate study areas to the north.

Again, a threshold was reached between the 1 m and 2 m SLR scenarios for several of the coastal ecosystems (S5 Table). Under the 2 m SLR scenario, SLAMM predicted that 15,428 ha of mangrove forest was lost or 64% of the current spatial extent. This is the opposite of the gains predicted under the slower rates of SLR examined. In addition, loss of saltmarsh and undeveloped dry land expanded greatly under the 2 m SLR scenario. Nearly all of the currently existing saltmarsh was predicted to be lost under this scenario (7,087 ha or 98%), and 15,635 ha (10%) of undeveloped dry land was predicted to be lost, more than double that under the 1 m SLR scenario. The predicted changes took place along most of the shorelines in the study area including the lower reaches of the three rivers emptying into Charlotte Harbor, the Caloosahatchee, the Peace and the Myakka; and the numerous islands.

Under the SLAMM scenarios with developed dry land allowed to transition (S5 Table), approximately 2%, 5%, and 23% of developed dry land was predicted to be lost under the 0.7 m, 1 m and 2 m scenarios, respectively. The direction and magnitude of change simulated for coastal wetlands were similar to the developed dry land protected scenarios under the 1 m SLR scenario with the exception that simulated changes in mangrove forest were much larger (+14,534 ha versus +8,251 ha), ocean beach expanded instead of declined (+139 ha versus -201 ha), and estuarine beach expanded to a greater extent (+309 ha versus +38 ha).

The spatial results for all sites and SLR scenarios modeled have been posted for viewing on Coastal Resilience 2.0, a suite of map based, decision-making tools for coastal risk assessment available on the web (coastalresilience.org).

### Summary of Results, All Sites

**Fig 9:**
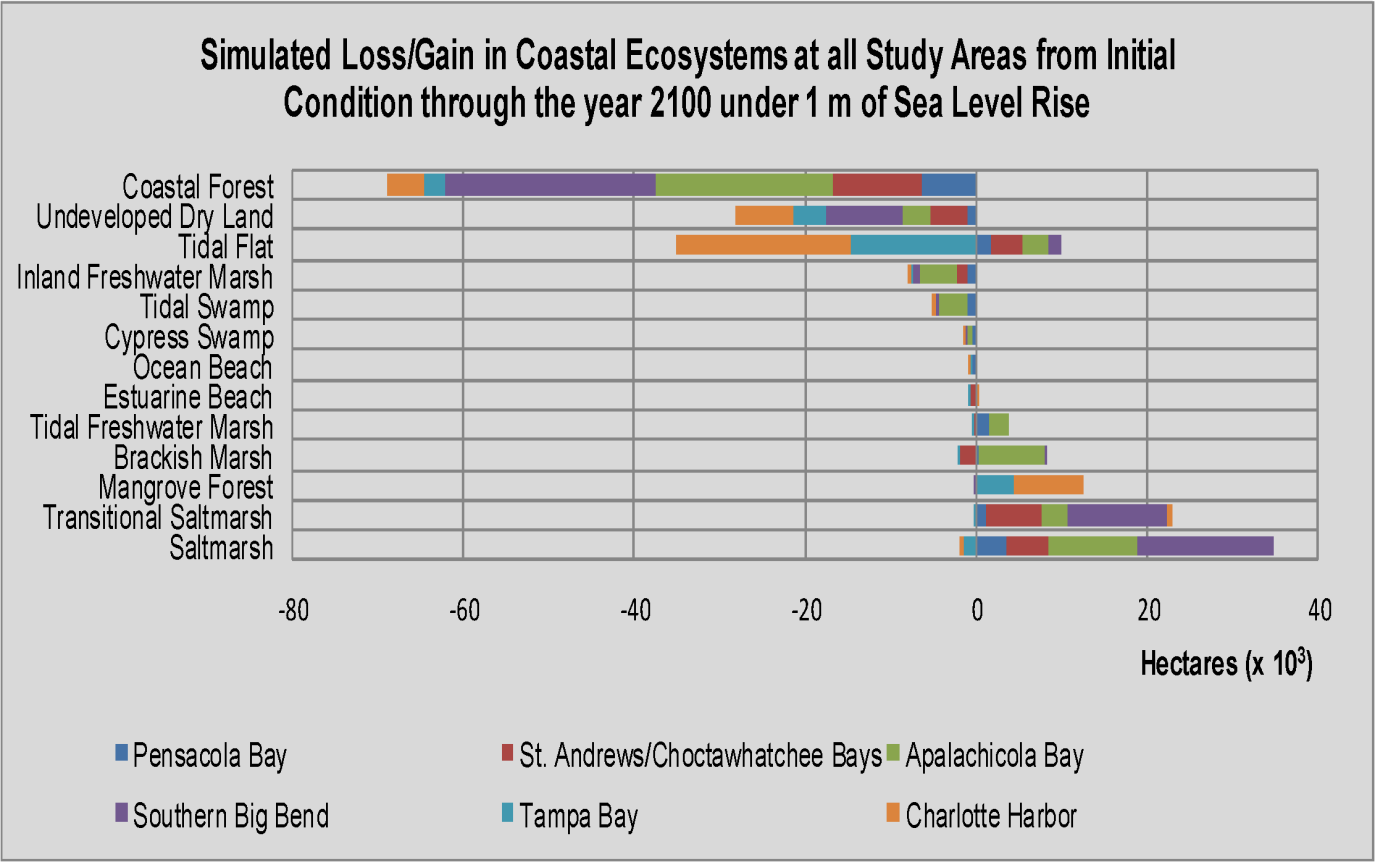
Bar graph of loss/gain of coastal ecosystems for all six study areas (summed). The SLAMM results illustrated in this bar graph are for the following 3 sea level rise scenarios: 0.7m, 1m, 2m. All scenarios were run with developed dry land protected from change in the SLAMM user interface.

Overall, coastal wetland ecosystems at all six study areas along Florida’s Gulf Coast are likely to change substantially. Under the 1 m SLR scenario, SLAMM predicted that coastal forest, tidal flat, inland freshwater marsh and tidal swamp will lose the most spatial extent, -69,309 ha, -25,552 ha, -7,733 ha, and -5,069 ha, respectively (Table 4; Fig 9). In terms of percent loss, the coastal wetland ecosystems predicted to be most adversely affected included tidal swamp, tidal flat, coastal forest and estuarine beach with -54%, -47%, -18% and -17%, respectively. In addition, undeveloped dry land was predicted by SLAMM to lose -28,445 ha under the 1 m SLR scenario, or 2% of the developed dry land in the study areas. Coastal wetland ecosystems predicted by SLAMM to gain spatial extent under the 1 m SLR scenario included saltmarsh (+32,923 ha; 88%), transitional saltmarsh (+23,645 ha; 81%), mangrove forest (+12,583 ha; 60%), brackish marsh (+6,365 ha; 40%) and tidal freshwater marsh (+3,594 ha). As was noted for some of the individual study areas, some reversals were noted in habitat responses between the 1 m and 2 m SLR by 2100 scenarios (Fig 9). For example, at the slower rates of SLR studied (0.7 m and 1 m by 2100), mangrove forests were able to expand in extent, but at 2 m of SLR by 2100, they lost extent to open water. The same threshold seemed to be acting on estuarine beach and ocean beach as both these ecosystems more than doubled in percent lost between the 1 m and 2 m SLR by 2100 scenarios.

**Table 4:** Change in coastal wetland ecosystems and adjacent dry land for the six study areas combined under 3 sea level rise scenarios, developed dry land protected from changing.

All Study Areas		0.7m		1 m		2 m	
Coastal Ecosystem	Initial Condition	Change (ha)	Percent Change	Change (ha)	Percent Change	Change (ha)	Percent Change
**Undeveloped Dry Land**	1,172,423	-18,071	-2%	-28,445	-2%	-66,523	-6%
**Coastal Forest**	392,433	-56,447	-14%	-69,309	-18%	-98,924	-25%
**Tidal Flat**	54,069	-27,016	-50%	-25,552	-47%	2,724	5%
**Inland Freshwater Marsh**	124,373	-4,837	-4%	-7,733	-6%	-15,062	-12%
**Tidal Swamp**	9,471	-3,490	-37%	-5,069	-54%	-4,143	-44%
**Cypress Swamp**	33,496	-887	-3%	-1,303	-4%	-2,484	-7%
**Ocean Beach**	5,195	-851	-16%	-692	-13%	-1,832	-35%
**Estuarine Beach**	2,010	-395	-20%	-341	-17%	-729	-36%
**Tidal Freshwater Marsh**	4,418	6,563	149%	3,594	81%	1,692	38%
**Brackish Marsh**	10,652	6,944	65%	6,365	60%	3,224	30%
**Mangrove Forest**	31,230	11,014	35%	12,583	40%	-18,272	-59%
**Transitional Salt Marsh**	114	29,855	na^1^	23,645	na^1^	32,672	na^1^
**Saltmarsh**	37,379	20,100	54%	32,923	88%	4,689	13%
**NET For ALL Wetlands**	704,839	-19,447	-3%	-30,890	-4%	-96,447	-14%

^1^No transitional saltmarsh was present in the initial condition so percent change cannot be calculated.

Under the SLAMM scenarios with developed dry land allowed to transition, approximately 2%, 4% and 15% of developed dry land were predicted to be lost under the 0.7 m, 1 m and 2 m scenarios, respectively (S6 Table). The direction and magnitude of change simulated for coastal wetlands were similar to the developed dry land protected scenarios under the 1 m SLR scenario with the exceptions that many more hectares of mangrove forest and estuarine beach were gained versus lost (26,318 ha versus 12,583 ha and +506 ha versus -341 ha, respectively). Overall there was a modest increase in wetlands of all types rather than a modest loss (+29,534 ha versus -30,890 ha).

### Uncertainty Analyses

Uncertainty results were characterized by subtracting the minimum output from the maximum output values (Table 5). A scale was created to simplify characterization of the uncertainty results as follows: difference over 10,000 ha, uncertainty large; difference between 1,000 and 10,000 ha, uncertainty moderate; difference between 100 and 1,000 ha, uncertainty modest; difference between 1 and 100, uncertainty low; difference less than 1, no uncertainty.

**Table 5:** Uncertainty Analysis on selected numerical input parameters at three study areas.

Study Area	PENS	PENS	SBB	SBB	TAM
Distribution Type	Uniform	Triangular	Triangular	Uniform	Multi
Distribution Parameter	Brackish Marsh Accr	Saltmarsh Accr	Saltmarsh Accr	Sedimentation	Multi
Variable Name	Max-Min	Max-Min	Max-Min	Max-Min	Max-Min
**Developed Dry Land**		0.1	0.8	0.8	2035.1
**Undeveloped Dry Land**		0	0.6	0.3	483.7
**Coastal Forest**		0	2.4	0.6	195.2
**Cypress Swamp**		0	0	0	1.7
**Inland Freshwater Marsh**	1.2	0	0	0	51.8
**Tidal Freshwater Marsh**	0	0	0	0	0
**Transitional Saltmarsh**	103.2	0.7	9.1	0.7	28.4
**Saltmarsh**	144.5	297.1	26462.5	1.4	1554.6
**Mangrove**	0	0	0.1	0	2591.5
**Estuarine Beach**	1.3	0	32.2	2.4	53.8
**Tidal Flat**	186.3	158	16775	126.6	1829.5
**Ocean Beach**	2.2	0	1.2	2.4	222
**Brackish Marsh**	333.7	0	0	0	0
**Tidal Swamp**	0	0	0	0	0

PENS = Pensacola Bay; SBB = Southern Big Bend; TAM = Tampa Bay; Accr = accretion.

The uncertainty analysis for the Pensacola Bay Study Area was conducted on only a portion of the study area (87,796 ha versus 351,679 ha) due to the extremely long runtime of the analysis on the entire site. At the Pensacola Bay study area, the uncertainty analyses on brackish marsh accretion and saltmarsh accretion revealed only small to modest changes in the 2100 results for the various wetlands types with changes to the parameters as illustrated in Table 5. Based on these results, uncertainty in the results at the Pensacola Bay study area is low.

At the Southern Big Bend study area, the uncertainty analyses run on the two selected input parameters, saltmarsh accretion and sedimentation rate, revealed small to modest changes in the 2100 results for the various wetlands types with changes to the sedimentation rate (Table 5). However, large variations in saltmarsh and tidal flat results resulted when saltmarsh accretion rate was varied and saltmarsh results varied from a low of 22,216 ha to a high of 48,686 ha with a greater frequency of return at the higher end of the range. SLAMM predicted 48,584 ha, which is at the higher end of the range. Tidal flat results also returned high variation when an uncertainty analysis was run on saltmarsh accretion rate. Tidal flat results varied from a low of 5,616 ha to a high of 22,396 ha with a greater frequency of return at the lower end of the range. SLAMM predicted 5,345 ha which is at the lower end of the range. Based on these results, uncertainty in the results at the Big Bend study area for saltmarsh and tidal flat would have been considered high, except that a hindcast of SLAMM on a portion of this study area showed a close match with more than 20 years of field studies [30]. Since change in saltmarsh extent in the face of SLR is directly tied to saltmarsh accretion rate over time, it is not surprising that the uncertainty results indicated that changes in saltmarsh and tidal flat are sensitive to saltmarsh accretion rate. The availability of empirically derived saltmarsh accretion rates could improve the precision of SLAMM projections of how SLR will affect coastal wetlands.

The uncertainty analysis for the Tampa Bay study area was run on three input parameters: salt elevation, saltmarsh accretion rate and beach sedimentation rate. This analysis revealed low to moderate changes in the 2100 results for the various wetland and dry land types with changes to the selected input parameters (Table 5). Based on these results, uncertainty in the results at this site were moderate. These results indicated that improving the accuracy of saltmarsh accretion, beach sedimentation and salt elevation data available for the Tampa Bay study area could moderately improve projections of how SLR will affects coastal wetlands in the study area.

Overall, the uncertainty results identified small to moderate concerns over the precision of the SLAMM results. The greatest uncertainty arose in parameters directly tied to the response of coastal ecosystems to SLR such as with marsh accretion rates. Precision uncertainty is greatly diminished at sites where long-term studies of coastal ecosystem accretion, sedimentation and erosion have been conducted.

## Discussion and Conclusions

These results illustrate the utility of SLAMM for quantitatively, qualitatively and spatially describing how coastal wetland ecosystems and adjacent dry land areas may change as a result of predicted changes in SLR and how this information can be used to develop and prioritize adaptation strategies. The spatial extent of the change varies with the coastal habitat type, elevation of upland areas, the rate of SLR and other wetland processes. The uncertainty analyses highlighted the need for collecting long-term site specific data on accretion, erosion and sedimentation to improve the predictive capabilities of SLAMM. While SLAMM predicts that one coastal ecosystem type will replace another ecosystem type under a particular SLR scenario, it gives no indication of how long the predicted transition may take. For example, SLAMM may report that a tidal swamp transitions into marsh. Field studies in the Waccasassa Bay area [49, 50] have shown that this transition can take decades depending on the plant species involved. Some of the potential increases in coastal wetland spatial extent are dependent on how adjacent upland areas are managed. If coastal wetlands are prevented from migrating to higher elevations as sea level rises, then loss in coastal wetland spatial extent will be much larger. Furthermore, predicted losses in coastal wetland extent may translate into gains in new shallow subtidal ecosystems (e.g., loss of tidal flat in the Tampa Bay and Charlotte Harbor study areas).

In addition to changes in spatial extent, the spatial orientation and relative percent coverages of coastal wetland ecosystems are predicted to change with SLR. Dependent species and adjacent human communities will be substantially affected if the predicted large-scale changes to coastal wetland ecosystems occur. Human communities located adjacent to these changed coastal wetland ecosystems may become more or less vulnerable to coastal storm impacts depending on the changes to coastal wetland ecosystems in their vicinity [51]. For example, mangrove and coastal forest cover can reduce the extent of storm surge and coastal flooding [22]. As a result, shifts in these systems will impact risk to developed areas. The Philippine city of Tacloban with little mangrove forest protection experienced some of the worst effects of Typhoon Haiyan [52]. As was seen in the Philippines following Typhoon Haiyan, human communities located near or behind coastal and mangrove forests were less likely to be damaged by wave energy following the 2004 the Indian Ocean tsunami [53]. Increased vulnerability to storm impacts can also affect property values, infrastructure investment, insurance rates and the local economy.

In addition to providing protection from coastal storms, coastal wetlands provide a number of other services, including erosion control, fish and wildlife habitat, recreation, and carbon sequestration. Changes in wetland ecosystem spatial extent and composition can affect all of these services. With respect to wildlife habitat, studies of Florida species likely to be vulnerable to the effects of SLR predicted that high percentages (21 to 39%) of the plant and animal species evaluated would likely suffer adverse effects or extinction from SLR as a result of habitat contraction [54]. These effects can, in turn, affect harvests (e.g., commercial fishing), recreational opportunities and tourism [55].

Spatial results can be used to identify promising locations for both protecting areas that will allow migration of coastal wetlands inland as sea level rises and restoring critical habitats that have been lost. The results can also be used to identify conservation priorities such as resilient wetland areas that are likely to persist despite sea level rise. Restoration can be based on where coastal wetlands are likely to become inundated, where coastal forests are likely to become marshes, or where undeveloped dry land is likely to become wetlands. Communities can use these results to assist with the development and siting of adaptation projects such as living shorelines, oyster reefs, wetland and upland habitat restoration, water flow preservation and sediment management to support vulnerable marsh, mangrove forest and dry land areas. A nature-based solution may be more cost effective and confer more benefits than an engineered solution to support shorelines [25, 56, 57].

The modeling results point to several potential adaptation strategies (see Table 6 for examples). In the study areas where large areas of coastal forest and/or tidal swamp were predicted to transition to marsh (Pensacola Bay, St. Andrews/ Choctawhatchee Bay, Apalachicola Bay and Southern Big Bend), planners and managers could seek additional opportunities to maintain or restore water and/or sediment flows. Where large areas of swamp and/or marsh were predicted to transition to open water (Pensacola Bay, St. Andrews/Choctawhatchee Bays, Apalachicola Bay, and Southern Big Bend), restoration of oyster reefs or other types of living shorelines at the existing marsh/open water interface could be used to slow this transition. Where large areas of undeveloped dry land were predicted to transition to mangrove forest such as in Charlotte Harbor, additional protection of these uplands could facilitate this transition and help maintain or enhance the storm protection qualities of this habitat. Installation of oyster reefs and living shorelines might also be valuable where large areas of tidal flat were predicted to transition to open water (Tampa Bay, Charlotte Harbor) to help reduce the rate of loss. Tidal flat restoration would also support species dependent on this ecosystem. As sea level rises, beaches are at risk of diminishing in size. While it would be cost prohibitive to renourish all barrier island beaches in the study areas, renourishment efforts could be prioritized by state and local governments using criteria such as areas most critical for protection of human populations and/or areas of greatest importance to harvested or vulnerable species such as sea turtles. The results of these analyses can also be used to inform the development of provisions or programs to discourage building on undeveloped lands that would otherwise become wetlands as sea level rises and/or to provide for the relocation of homes and businesses located in areas highly vulnerable to storm surge and rising sea level. These types of programs were implemented in New York State following the Super Storm Sandy [58].

**Table 6:** Examples of how the results of the SLR analyses can inform coastal resilience planning actions.

Project Area	SLR Result, 1 m by 2100	Potential Adaptation Action
**Pensacola Bay**	Large amounts of brackish marsh transitions to saltmarsh, tidal flat and open water in several areas.	Consider areas adjacent to open water for living shorelines to minimize or reduce the rate of transition. As a result, brackish marsh will persist longer for the benefit of dependent species.
**Pensacola Bay**	Ocean beaches are at risk of diminishing in extent as SLR rises.	Prioritize beach renourishment activities to maximize social and ecological benefits (e.g., tourism, sea turtle and shorebird nesting habitat).
**St. Andrews/ Choctawhatchee Bays**	Coastal forest transitions to marsh in several areas. One of these areas is adjacent to a developed area (Santa Rosa Beach).	Install/construct living shorelines to support brackish marsh and coastal forest in proximity to developed areas to maintain to their wind and wave energy reduction properties as long as possible.
**Apalachicola Bay**	A large area of freshwater marsh at the mouth of the Apalachicola River transitions to saltmarsh.	This area may be a good candidate for the installation of living shoreline habitat along the edge bordering open water to support the freshwater marsh and its floodwater absorption values in this area as long as possible.
**Southern Big Bend**	This entire area is likely to experience a large transition of coastal forest to saltmarsh.	Maintain freshwater flows to this coast to the greatest extent possible. Doing so will slow the transition of coastal wetland ecosystems.
**Tampa Bay**	There is a large area around the bay where wetlands and tidal flats transition to open water.	Install living shorelines where wetlands are transitioning to open water. Doing so will support these coastal wetland ecosystems as long as possible and will extend the life of the storm surge reduction benefits that they provide.
**Tampa Bay and Charlotte Harbor**	There are areas where undeveloped dry land transitions to mangrove forest.	Protect the land and shoreline where mangroves are predicted to expand and facilitate their expansion where needed to take advantage of wave attenuation and reduced flooding where mangroves are present (see text).
**Charlotte Harbor**	There are a few areas where mangrove forest transitions to open water.	These areas are good candidate sites for installing living shorelines to maintain the protective qualities of mangroves as long as possible.

Communities along Florida’s Gulf Coast and in other vulnerable areas that take action in the near term to implement adaptation strategies will have a longer planning horizon across which to spread adaptation costs and reap benefits. These communities will have a greater ability to mainstream adaptation into existing activities with the result of greatly increasing the affordability of these actions [59, 60]. Furthermore, the quantitative, qualitative and spatial information on coastal wetland change provided by this study can be used to assist in the development of monitoring programs to signal when on-the-ground changes are happening and at what rates. Future research focused on determining the most effective and cost efficient methods for implementing the potential adaptation strategies suggested in this study would be valuable at a global scale.

## Supporting Information

S1 Table Crosswalk between the Florida Cooperative Land Cover (CLCv1.1) and SLAMM vegetation categories. (PDF)

S2 Table Sources of Digital Elevation Data used in the SLAMM simulations. Study areas are listed from west to east.
(PDF)

S3 Table Numeric input parameters for each study area. (PDF)

S4 Table NOAA tide stations used in the SLAMM analysis for each project study area. (PDF)

S5 Table Coastal habitat change under 3 sea level rise scenarios for all study areas through the year 2100 where developed dry land is allowed to transition. (PDF)

S6 Table Change in coastal wetlands ecosystems and adjacent dry land for the six study areas under 3 sea level rise scenarios with developed dry land allowed to transition. The St. Andrews/Choctawhatchee Bay Results were provided by the Texas Chapter of The Nature Conservancy.
(PDF)

S1 Text Detailed Methods Used to Define the Numerical Input Parameters for Each Study Area and Study Area Subsite. (PDF)
