# Supplementary material for: Modeled Sea Level Rise Impacts on Coastal Ecosystems at Six Major Estuaries on Florida’s Gulf Coast: Implications for Adaptation Planning
Source: PLoS One. 2015 Jul 24;10(7):e0132079. doi: 10.1371/journal.pone.0132079 (PMC4514811; doi:10.1371/journal.pone.0132079)
Supplement: S1 Table — (PDF) [file pone.0132079.s001.pdf]

S1 Table. Crosswalk between the Florida Cooperative Land Cover (CLCv1.1) and SLAMM vegetation categories.

SLAMM Category	CLC Land Cover
Cypress Swamp	2210 - Cypress/Tupelo(incl Cypress/Tupelo mixed)
	2211 - Cypress
	2214 - Strand Swamp
Developed Dry Land	1821 - Low Intensity Urban
	1822 - High Intensity Urban
	1832 - Agriculture
	1840 - Transportation
	1841 - Roads
	1842 - Rails
	1850 - Communication
	1860 - Utilities
	1870 - Extractive
	1871 - Strip Mines
	1872 - Sand & Gravel Pits
	1873 - Rock Quarries
	1874 - Oil & Gas Fields
	1875 - Reclaimed Lands
	1877 - Spoil Area
	3240 - Sewage Treatment Pond
	3250 - Stormwater Treatment Areas
	3260 - Industrial Cooling Pond
	18211 - Urban Open Land
	18212 - Low Structure Density
	18221 - Residential, Med. Density - 2-5 Dwelling Units/AC
	18222 - Residential, High Density > 5 Dwelling Units/AC
	18223 - Commercial & Services
	18224 - Industrial
	18225 - Institutional
	18324 - Vineyard & Nurseries
	182111 - Urban Open Forested
	182112 - Urban Open Pine
	182131 - Parks
	182132 - Golf courses
	182135 - Cemeteries
	182136 - Community rec. facilities
Estuarine Water	3116 - Coastal Rockland Lake
	4160 - Tidally-influenced Stream
	5000 - Estuarine
Inland Freshwater	2100 - Freshwater Non-Forested Wetlands

SLAMM Category	CLC Land Cover
Marsh	
	2111 - Wet Prairie
	2112 - Mixed Scrub-Shrub Wetland
	2113 - Marl Prairie
	2120 - Freshwater Marshes
	2121 - Isolated Freshwater Marsh
	2122 - Coastal Interdunal Swale
	2123 - Floodplain Marsh
	2124 - Slough Marsh
	2125 - Glades Marsh
	2131 - Sawgrass
	2140 - Floating/Emergent Aquatic Vegetation
	2300 - Non-vegetated Wetland
	2410 - Impounded Marsh
	5251 - Buttonwood Forest
	21112 - Cutthroat Seep
	21121 - Shrub Bog
	21211 - Depression Marsh
	21212 - Basin Marsh
Inland Open Water	3100 - Natural Lakes & Ponds
	3111 - Clastic Upland Lake
	3112 - Coastal Dune Lake
	3113 - Flatwoods/Prairie/Marsh Lake
	3114 - River Floodplain Lake/Swamp Lake
	3115 - Sinkhole Lake
	3117 - Sandhill Lake
	3118 - Major Springs
	3200 - Artificial Lakes & Ponds
	3210 - Artificial/Farm Pond
	3211 - Aquacultural Ponds
	3220 - Artificial Impoundment/Reservoir
	3230 - Quarry Pond
	4100 - Natural Rivers & Streams
	4110 - Alluvial Stream
	4120 - Blackwater Stream
	4130 - Spring Run Stream
	4140 - Seepage Stream
	4200 - Canal/Ditch
	4210 - Canal
	8000 - Open Water
Inland Shore	4170 - Riverine Sandbar
Mangrove	5250 - Mangrove Swamp

SLAMM Category	CLC Land Cover
Ocean Beach	1610 - Beach Dune
	1670 - Sand Beach (Dry)
	6000 - Marine
Regularly Flooded Marsh	5240 - Saltwater Marsh
Rocky Intertidal	5230 - Oyster Bar
	52111 - Keys Tidal Rock Barren
Swamp	2141 - Slough
	2200 - Freshwater Forested Wetlands
	2213 - Isolated Freshwater Swamp
	2215 - Floodplain Swamp
	2220 - Other Coniferous Wetlands
	2221 - Wet Flatwoods
	2222 - Pond Pine
	2223 - Atlantic White Cedar
	2230 - Other Hardwood Wetlands
	2231 - Baygall
	2232 - Hydric Hammock
	2233 - Mixed Wetland Hardwoods
	2234 - Titi Swamp
	2240 - Other Wetland Forested Mixed
	2242 - Cypress/Pine/Cabbage Palm
	2420 - Impounded Swamp
	2450 - Wet Coniferous Plantations
	7400 - Exotic Wetland Hardwoods
	22131 - Dome Swamp
	22132 - Basin Swamp
	22211 - Hydric Pine Flatwoods
	22212 - Hydric Pine Savanna
	22311 - Bay Swamp
	22312 - South Florida Bayhead
	22321 - Coastal Hydric Hammock
	22322 - Prairie Hydric Hammock
	22323 - Cabbage Palm Hammock
	22331 - Bottomland Forest
	22332 - Alluvial Forest
	221311 - Stringer Swamp
	221312 - Gum Pond
Tidal Flat	5200 - Intertidal
	5220 - Tidal Flat
	9100 - Unconsolidated Substrate
Tidal Fresh Marsh	21231 - Freshwater Tidal Marsh

SLAMM Category	CLC Land Cover
Tidal Swamp	22151 - Freshwater Tidal Swamp
Undeveloped Dry Land	1110 - Upland Hardwood Forest
	1111 - Dry Upland Hardwood Forest
	1112 - Mixed Hardwoods
	1120 - Mesic Hammock
	1122 - Prairie Mesic Hammock
	1123 - Live Oak
	1124 - Pine - Mesic Oak
	1125 - Cabbage Palm
	1130 - Rockland Hammock
	1131 - Thorn Scrub
	1140 - Slope Forest
	1150 - Xeric Hammock
	1210 - Scrub
	1211 - Oak Scrub
	1213 - Sand Pine Scrub
	1214 - Coastal Scrub
	1220 - Upland Mixed Woodland
	1230 - Upland Coniferous
	1231 - Upland Pine
	1240 - Sandhill
	1311 - Mesic Flatwoods
	1312 - Scrubby Flatwoods
	1320 - Pine Rockland
	1330 - Dry Prairie
	1400 - Mixed Hardwood-Coniferous
	1410 - Successional Hardwood Forest
	1500 - Shrub and Brushland
	1620 - Coastal Berm
	1630 - Coastal Grassland
	1640 - Coastal Strand
	1650 - Maritime Hammock
	1660 - Shell Mound
	1710 - Sinkhole
	1720 - Upland Glade
	1740 - Keys Cactus Barren
	1811 - Vegetative Berm
	1831 - Rural Open
	1880 - Bare Soil/Clear Cut
	2114 - Seepage Slope
	7000 - Exotic Plants
	7100 - Australian Pine

SLAMM Category	CLC Land Cover
	7200 - Melaleuca
	7300 - Brazilian Pepper
	18311 - Rural Open Forested
	18312 - Rural Open Pine
	18321 - Cropland/Pasture
	18322 - Orchards/Groves
	18323 - Tree Plantations
	183111 - Oak - Cabbage Palm Forests
	183211 - Row Crops
	183212 - Field Crops
	183213 - Improved Pasture
	183214 - Unimproved/Woodland Pasture
	183221 - Citrus
	183222 - Fruit Orchards
	183224 - Fallow Orchards
	183231 - Hardwood Plantations
	183232 - Coniferous Plantations
	183241 - Tree Nurseries
	183242 - Sod Farms
	183243 - Ornamentals
	183245 - Floriculture
	183251 - Feeding Operations
	183252 - Specialty Farms
	222111 - Cutthroat Grass Flatwoods
	1832121 - Sugarcane
	1832151 - Fallow Cropland
