# Supplementary material for: Modeled Sea Level Rise Impacts on Coastal Ecosystems at Six Major Estuaries on Florida’s Gulf Coast: Implications for Adaptation Planning
Source: PLoS One. 2015 Jul 24;10(7):e0132079. doi: 10.1371/journal.pone.0132079 (PMC4514811; doi:10.1371/journal.pone.0132079)
Supplement: S2 Table — Study areas are listed from west to east. (PDF) [file pone.0132079.s002.pdf]

S2 Table. Sources of Digital Elevation Data used in the SLAMM simulations. Study areas are listed from west to east.

Study Area	DEM Source Dataset	DEM Source
Pensacola Bay	2006 Escambia, Santa Rosa, and Walton Counties LiDAR	NOAA Coastal Services Center website
St. Andrews/ Choctawhatchee Bays	2004 US Army Corps of Engineers (USACE) Post-Hurricane Ivan Topo/Bathy LiDAR for Alabama and Florida; 2005 USACE Post-Hurricane Dennis Topo/Bathy LiDAR Project, Alabama and Florida Coasts; 2006 Florida LiDAR: Escambia, Santa Rosa, and Walton Counties; 2007 Florida Division of Emergency Management (FDEM) LiDAR for Bay, Gulf and coastal Okaloosa counties; 2007 Northwest Florida Water Management District (NWFWMD) LiDAR for portions of Bay, Calhoun, Jackson and Washington Counties; 2008 NWFWMD LiDAR for Eglin Air Force Base, Walton	
Apalachicola Bay	Franklin and Bay County 2007 FL Division of Emergency Management LiDAR	FL Division of Emergency Management
Southern Big Bend	Tiled DEM data (from 2006-2007 LiDAR collections)	Southwest Florida Water Management District
	Pasco County 2004-2008 FL Division of Emergency Management: Southwest Florida LiDAR	NOAA Coastal Services Center website
	NED 1/3 arc-second (small area to fill in missing marsh)	USGS National Map website
Tampa Bay	TB_DEM_10m_m	Tampa Bay Estuary Program (TBEP)
Charlotte Harbor	2005 SWFWMD Peace River South District LiDAR<br>2004-2008 Florida Division of Emerg. Mgmt.: SW Florida LiDAR	NOAA Coastal Services Center website
