# Supplementary material for: Modeled Sea Level Rise Impacts on Coastal Ecosystems at Six Major Estuaries on Florida’s Gulf Coast: Implications for Adaptation Planning
Source: PLoS One. 2015 Jul 24;10(7):e0132079. doi: 10.1371/journal.pone.0132079 (PMC4514811; doi:10.1371/journal.pone.0132079)
Supplement: S3 Table — (PDF) [file pone.0132079.s003.pdf]

S3 Table. Numeric input parameters for each study area.

Study Area	PEN	PEN	PEN	SAC	SAC	SAC	APA	APA	SBB	SBB	SBB	SBB	SBB	TAM	TAM	TAM	CH	CH	CH	CH	CH
**Parameter (Global= G; Subsite =S)**	G	S1	S2	S1	S2	S3	G	S1	G	S1	S2	S3	S4	G	S1	S2	G	S1	S2	S3	S4
**Land cover photo date**	‘06	‘06	‘06	‘04	‘04	‘04	‘06	‘06	‘08	‘08	‘08	‘08	‘08	‘07	‘07	‘07	‘04	‘08	‘04	‘04	‘08
**DEM Date**	‘06	‘06	‘07	‘07	‘07	‘07	‘07	‘07	‘07	‘07	‘07	‘07	‘07	‘07	‘07	‘07	‘07	‘05	‘07	‘07	‘07
**Direction Offshore [n,s,e,w]**	S	S	S	S	S	S	S	S	W	W	W	W	W	W	W	W	W	S	W	W	W
**Historic trend in SLR (mm/yr)**	2.1	2.1	2.1	0.75	0.75	0.75	1.38	1.38	1.8	1.8	1.8	1.8	1.8	2.43	2.43	2.36	2.4	2.4	2.4	2.4	2.4
**NAVD correction [MTL - NAVD88 (m)]**	[a]	[a]	[a]	[a]	[a]	[a]	-0	0.06	[a]	[a]	[a]	[a]	[a]	-0.14	-0.09	-0.08	-0.18	-0.14	-0.19	-0.13	-0.17
**Great diurnal tide range (m)**	0.41	0.47	0.38	0.38	0.16	0.43	0.67	0.47	1.19	0.59	0.92	0.59	1.19	0.67	0.87	0.79	0.53	0.62	0.75	0.40	0.46
**Salt elevation (m above MTL)**	0.54	0.47	0.57	0.5	0.26	0.61	0.59	0.22	0.9	0.49	0.72	0.48	0.9	0.55	0.71	0.65	0.43	0.51	0.62	0.53	0.38
**Marsh erosion rate (horz. m/yr)**	2[b]	2[b]	2[b]	0.83[f]	0.83[f]	0.83[f]	2[b]	2[b]	0.32[h]	0.32[h]	0.32[h]	0.32[h]	0.32[h]	2[b]	2[b]	2[b]	0.23[h]	0.23[h]	0.23[h]	0.23[h]	0.23[h]

**Coastal forest erosion rate (horz. m/yr)**	1[b]	1[b]	1[b]	1[b]	1[b]	1[b]	1[b]	1[b]	0.32[h]	0.32[h]	0.32[h]	0.32[h]	0.32[h]	1[b]	1[b]	1[b]	0.23[h]	0.23[h]	0.23[h]	0.23[h]	0.23[h]
**Tidal flat erosion rate (horz. m/yr)**	0.5[b]	0.5[b]	0.5[b]	0.5[b]	0.5[b]	0.5[b]	0.5[b]	0.5[b]	0.32[h]	0.32[h]	0.32[h]	0.32[h]	0.32[h]	0.5[b]	0.5[b]	0.5[b]	0.7[k]	0.13[k]	0.7[k]	0.13[k]	0.13[k]
**Saltmarsh accretion rate (mm/yr)**	2.25[c]	2.25[c]	2.25[c]	4.9[g]	4.9[g]	4.9[g]	0.8	0.8	7.2[i]	7.2[i]	7.2[i]	7.2[i]	7.2[i]	2.25[c]	2.25[c]	2.25[c]	7.2[l]	7.2[l]	7.2[l]	7.2[l]	7.2[l]
**Brackish marsh accretion rate (mm/yr)**	3.75[c]	3.75[c]	3.75[c]	4.9[g]	4.9[g]	4.9[g]	1.2	1.2	7.2[i]	7.2[i]	7.2[i]	7.2[i]	7.2[i]	3.75[c]	3.75[c]	3.75[c]	7.2[l]	7.2[l]	7.2[l]	7.2[l]	7.2[l]
**Tidal freshwater marsh accr. rate (mm/yr)**	4[c]	4[c]	4[c]	5.9[g]	5.9[g]	5.9[g]	1.45	1.45	7.2[i]	7.2[i]	7.2[i]	7.2[i]	7.2[i]	4[c]	4[c]	4[c]	7.2[l]	7.2[l]	7.2[l]	7.2[l]	7.2[l]
**Inland-Fresh Marsh Accr (mm/yr.)**	na	na	na	5.9[g]	5.9[g]	5.9[g]	na	na	na	na	na	na	na	na	na	na	na	na	na	na	na
**Mangrove Accr (mm/yr.)**	na	na	na	7[g]	7[g]	7[g]	na	na	na	na	na	na	na	na	na	na	na	na	na	na	na
**Tidal Swamp Accr (mm/yr.)**	na	na	na	1.1[g]	1.1[g]	1.1[g]	na	na	na	na	na	na	na	na	na	na	na	na	na	na	na
**Swamp Accretion (mm/yr.)**	na	na	na	0.3[g]	0.3[g]	0.3[g]	na	na	na	na	na	na	na	na	na	na	na	na	na	na	na

**Beach sedimentation rate (mm/yr)**	0.5[c]	0.5[c]	0.5[c]	0.5[c]	0.5[c]	0.5[c]	0	0	0.5[c]	0.5[c]	0.5[c]	0.5[c]	0.5[c]	2.7[j]	2.7[j]	2.7[j]	0.3[m]	0.3[m]	0.3[m]	0.3[m]	0.3[m]
**Frequency of overwash (years)**	25[b]	25[b]	25[b]	25[b]	25[b]	25[b]	25[b]	25[b]	25[b]	25[b]	25[b]	25[b]	25[b]	0[d]	0[d]	0[d]	31[n]	31[n]	31[n]	31[n]	31[n]
**Used elevation pre-processor [True=T, False=F]**	F	F	F	F	F	F	F	F	F	F	F	T	T	F	F	F	F	F	F	F	F

[a] Vdatum program (vdatum.noaa.gov) was used to create a raster for the correction.
[b]No site specific erosion rates were available from published literature, so the default SLAMM values from the Technical Documentation were used. Clough, J.S., 2012. SLAMM 6.2 Technical Documentation. Warren Pinnacle Consulting, Inc.,
[c]No site specific accretion or sedimentation rates were available from published literature, so values from Clough 2006 were used.
[d]Overwash function was turned off.
[e]Stapor FW. 1971. Sediment budgets on a compartmented low-to-moderate energy coast in northwest Florida. Marine Geology 10:M1-M7.
[f]Kish S. 2011. "Erosion Data" as reported in Clough J. 2011. Application of the Sea-Level Affecting Marshes Model (SLAMM 6) to Saint Andrew and Choctawhatchee Bays. Report prepared for The Nature Conservancy by Warren Pinnacle Consulting, Inc.
[g]Clough J. 2011. Application of the Sea-Level Affecting Marshes Model (SLAMM 6) to Saint Andrew and Choctawhatchee Bays. Report prepared for The Nature Conservancy by Warren Pinnacle Consulting, Inc
[h]Hine AC and DF Belknap. 1986. Recent geological history and modern sedimentary processes of the Pasco, Hernando, and Citrus County coastline: west central Florida: Florida Sea Grant College Report No. 79.
[i]Leonard LA, Hine AC, Luther ME. 1995. Surficial Sediment Transport and Deposition Processes in a Juncus roemerianus Marsh, Journal of Coastal Research, Vol. 11, No. 2, 15 pp. 322-336.
[j]Brooks, GR. 1989. Distribution Patterns and Accumulation Rates of Fine-Grained Sediments in Upper Tampa Bay, Florida Reports. Paper 85. http://scholarcommons.usf.edu/basgp_report/85
[k]Morton R, T Miller and L Moore. National Assessment of Shoreline Change, Part 1: Historical Shoreline Changes and Associated Coastal Land Loss Along the U.S. Gulf of Mexico. U.S. Geological Survey Open-file Report 2004-1043, http://pubs.usgs.gov/of/2004/1089/.
[l]Leonard LA, Hine AC, Luther ME. 1995. Surficial Sediment Transport and Deposition Processes in a Juncus roemerianus Marsh, Journal of Coastal Research, Vol. 11, No. 2, 15 pp. 322-336.
[m]Scholl D, F Craidhead, Sr., M Stuiver. 1969. Florida submergence curve revised: Its relation to coastal sedimentation rates. Science 163(3867):562–564, DOI: 10.1126/science.163.3867.562.

[n]Since 1888, four category 2 or higher hurricanes have passed through the area, so on average an overwash event has occurred approximately once every 31 years over the 123 year period of record (1888 to 2011; NOAA's Coastal Service Center website http://www.csc.noaa.gov.hurricanes).
