# Supplementary material for: Modeled Sea Level Rise Impacts on Coastal Ecosystems at Six Major Estuaries on Florida’s Gulf Coast: Implications for Adaptation Planning
Source: PLoS One. 2015 Jul 24;10(7):e0132079. doi: 10.1371/journal.pone.0132079 (PMC4514811; doi:10.1371/journal.pone.0132079)
Supplement: S4 Table — (PDF) [file pone.0132079.s004.pdf]

S4 Table. NOAA tide stations used in the SLAMM analysis for each project study area.

Study Area	Station	Station ID
Pensacola Bay	Navarre Beach	8729678
	Santa Rosa Sound East End	8729679
	East Bay Holley	8729702
	Woodlawn Beach	8729736
	Shield Point	8729747
	Blackwater River	8729753
	Hernandez Point North	8729791
	Fishing Bend	8729806
	Lora Point	8729816
	Floridatown	8729824
	Pensacola	8729840
	Bayou Chico	8729849
	Millview Perdido Bay	8729905
	Big Lagoon	8729909
	Blue Angels Park	8729941
	Hurst Hammock Perdido River	8729943
	Nix Point	8729962
	Perdido Key Old River	8729974
St. Andrews/Choctawhatchee Bays	ST. JOSEPH POINT, ST. JOSEPH BAY, FL	8728958
	WETAPPO CREEK, EAST BAY	8728973
	ALLANTON, EAST BAY	8729015
	SOUTHPORT, NORTH BAY, FL	8729017
	PARKER BRANCH, LAIRD BAYOU, EAST BAY, FL	8729039
	LAIRD BAYOU, EAST BAY, FL	8729045
	CALLAWAY BAYOU, EAST BAY, FL	8729063
	MILL BAYOU, NORTH BAY, FL	8729071
	DUPONT BRIDGE, EAST BAY, FL	8729083
	PARKER, EAST BAY, FL	8729084
	PEARL BAYOU, EAST BAY, FL	8729085
	SOUTHPORT, NORTH BAY, FL	8729101
	LYNN HAVEN, NORTH BAY, FL	8729102
	BEACON BEACH, ST ANDREW BAY, FL	8729105
	PANAMA CITY, ST. ANDREW BAY, FL	8729108
	SHELL ISLAND, ST. ANDREWS BAY, FL	8729119
	NEW ENTRANCE CHANNEL, ST. ANDREW BAY, FL	8729136
	ST. ANDREWS STATE PARK, GRAND LAGOON, FL	8729141
	ST. ANDREW STATE PARK, FL	8729149

	ALLIGATOR BAYOU, PANAMA CITY, FL	8729152
	BURNT MILL CREEK, WEST BAY, FL	8729154
	GRAND LAGOON, WEST END, FL	8729155
	SHELL POINT, WEST BAY, FL	8729169
	CROOKED CREEK, WEST BAY, FL	8729179
	PANAMA CITY BEACH, FL	8729189
	PANAMA CITY BEACH, GULF OF MEXICO, FL	8729210
	CHOCTAWHATCHEE RIVER, FL	8729329
	JOLLY BAY, CHOCTAWHATCHEE BAY, FL	8729332
	LA GRANGE BAYOU, FL	8729333
	ALLAQUAY BAYOU, FL	8729364
	SANTA ROSA HOGTOWN BAYOU, FL	8729376
	BASIN CREEK, FL	8729381
	BIG HAMMOCK PT, CHOCTAWHATCHEE BAY, FL	8729435
	VALPARISO, BOGGY BAYOU, FL	8729501
	VALPARAISO	8729501
	DESTIN, EAST PASS, FL	8729511
	GARNIER BAYOU, SHALIMAR	8729538
	CAMP PINCHOT, FL	8729548
	CAMP PINCHOT	8729548
	FORT WALTON BEACH, SANTA ROSA SOUND, FL	8729554
	CINCO BAYOU, FL	8729567
	HULBERT FIELD, FL	8729598
	HARRIS, SANTA ROSA SOUND, FL	8729613
Apalachicola Bay	Ne End St George Island	8728486
	South Carrabelle Beach	8728488
	White Beach East Bay	8728694
	Cat Point Apalachicola Bay	8728619
	St. George Island Bayside	8728626
	Apalachicola	8728690
	Sikes Cut St George Island	8728669
	Apalachicola River	8728711
	Huckleberry Landing Jackson River	8728757
	Eleven Mile St Vincent Sound	8728786
Southern Big Bend	Hudson Hudson Creek	8727061
	Fillmans Bayou	8727086
	Aripeka	8727097
	Bayport	8727151
	Johns Island Chassahowitzka Bay	8727235
	Chassahowitzka River	8727246

	Mason Creek Homosassa Bay	8727274
	Tuckers Island Homosassa River	8727277
	Halls River Bridge Halls River	8727293
	Ozello	8727306
	Ozello North	8727328
	Mangrove Point	8727333
	Dixie Bay	8727336
	Crystal River Kings Bay	8727343
	Twin Rivers Marina	8727348
	Shell Island Crystal River	8727359
	Port Inglis Withlacoochee River	8727395
	Yankeetown Withlacoochee River	8727411
	Waccasassa River	8727471
	Cedar Key	8727520
Tampa Bay	CORTEZ	8726217
	PERICO ISLAND	8726232
	ANNA MARIA OUTSIDE	8726243
	BRADENTON, MANATEE RIVER	8726247
	PALMA SOLA BAY NORTH	8726249
	DESOTO POINT	8726273
	ANNA MARIA CITY PIER	8726282
	EGMONT KEY, TAMPA BAY	8726347
	MULLET KEY, TAMPA BAY	8726364
	Port Manatee	8726384
	TIERRA VERDE	8726428
	LITTLE MANATEE RIVER	8726436
	MARSH BRANCH, RUSKIN	8726467
	MANGROVE POINT INSIDE	8726492
	St. Petersburg	8726520
	JOHNS PASS	8726533
	APOLLO BEACH, HILLSBOROUGH BAY	8726537
	NEWMAN BRANCH	8726539
	Old Port Tampa	8726607
	ALAFIA RIVER NORTH	8726614
	ARCHIE CREEK	8726632
	TAMPA, BALLAST POINT	8726639
	GANDY BRIDGE, OLD TAMPA BAY	8726641
	DAVIS ISLAND, HILLSBOROUGH BAY	8726657
	Mckay Bay Entrance	8726667
	TAMPA, HOOKER POINT, HILLSBOROUGH BAY	8726668
	TWENTY-SECOND ST. CAUSEWAY	8726685

	Bay Aristocrat Village	8726689
	CLEARWATER, CLEARWATER HARBOR	8726706
	WEST TAMPA, HILLSBOROUGH RIVER	8726711
	Clearwater Beach	8726724
	SAFETY HARBOR, OLD TAMPA BAY	8726738
	DUNEDIN CITY DOCK	8726761
	HONEYMOON ISLAND, SOUTH	8726808
	ANCLOTE RIVER	8726924
Charlotte Harbor	Bokellia	8725541
	El Jobean	8725769
	Englewood Lemon Bay	8725747
	Fort Myers	8725520
	Hendry Creek	8725377
	Liverrpool	8725835
	Locust Pt Hog Island	8725745
	Myakka River US 41	8725837
	Nokomis Venice Inlet	8725899
	North Captiva Island	8725488
	Ostego Bay	8725331
	Pine Island	8725528
	Placida Gasparilla Sound	8725667
	Punta Gorda	8725744
	Punta Rassa	8725391
	Tarpon Bay	8725362
	Turtle Bay	8725649
	Matanzas Pass Estero Island	8725366
	Hurricane Bay San Carlos Island	8725368
	Estero Island Estero Bay	8725351
	Coconut Point Estero Bay	8725319
	Port Boca Grande Charlotte Hbr	8725577
	Manasota	8725809
	Shakett Creek	8725902
	Little Gasparilla Island	8725665
	South Lemon Bay	8725691
	Harbour Heights	8725791
	Shell Creek Seaboard Rr	8725782
	Shell Creek Peace River	8725781
	Cutoff South	8725685
	Estero River Estero Bay	8725346
