# Supplementary material for: Modeled Sea Level Rise Impacts on Coastal Ecosystems at Six Major Estuaries on Florida’s Gulf Coast: Implications for Adaptation Planning
Source: PLoS One. 2015 Jul 24;10(7):e0132079. doi: 10.1371/journal.pone.0132079 (PMC4514811; doi:10.1371/journal.pone.0132079)
Supplement: S5 Table — (PDF) [file pone.0132079.s005.pdf]

S5 Table. Coastal habitat change under 3 sea level rise scenarios for all study areas through the year 2100 where developed dry land is allowed to transition.

SLR Scenario		0.7 m			1 m			2 m		
**Coastal Ecosystem**	**Initial Condition (ha)**	**2100 (ha)**	**2100-IC (ha)**	**Percent Change**	**2100 (ha)**	**2100-IC (ha)**	**Percent Change**	**2100 (ha)**	**2100-IC (ha)**	**Percent Change**
**Pensacola Bay Study Area**										
Undeveloped Dry Land	101097	100430	-667	-1%	100026	-1071	-1%	98463	-2634	-3%
Developed Dry Land	47506	47034	-472	-1%	46648	-858	-2%	45019	-2487	-5%
Coastal Forest	35695	30533	-5162	-15%	29286	-6408	-18%	25394	-10301	-29%
Inland Freshwater Marsh	9733	9034	-699	-7%	8685	-1048	-11%	7647	-2086	-21%
Brackish Marsh	3121	4865	1745	56%	3502	381	12%	2515	-606	-19%
Tidal Swamp	2637	2216	-422	-16%	1654	-983	-37%	1937	-700	-27%
Ocean Beach	2241	1940	-302	-14%	1888	-353	-16%	1333	-908	-41%
Cypress Swamp	1311	1006	-305	-23%	941	-370	-28%	762	-548	-42%
Saltmarsh	120	1738	1618	na[1]	3916	3796	na[1]	6233	6112	na[1]
Tidal Flat	90	568	478	na[1]	1916	1826	na[1]	3546	3456	na[1]
Tidal Freshwater Marsh	46	1488	1442	na[1]	1578	1532	na[1]	1915	1869	na[1]
Transitional Salt Marsh	31	1592	1561	na[1]	1670	1639	na[1]	3012	2981	na[1]
**Apalachicola Bay**										
Undeveloped Dry Land	57154	55491	-1664	-3%	54071	-3084	-5%	47140	-10014	-18%
Coastal Forest	77456	59760	-17696	-23%	56598	-20858	-27%	48765	-28690	-37%
Inland Freshwater Marsh	22182	19045	-3137	-14%	17804	-4379	-20%	16112	-6070	-27%
Tidal Swamp	5999	3552	-2447	-41%	2642	-3356	-56%	3381	-2618	-44%
Tidal Freshwater Marsh	3836	9036	5200	136%	6139	2303	60%	4194	358	9%
Brackish Marsh	2909	8740	5831	200%	10743	7834	269%	11128	8219	283%
Cypress Swamp	2651	2216	-435	-16%	2007	-644	-24%	1648	-1003	-38%
Developed Dry Land	2635	2500	-135	-5%	2397	-238	-9%	1965	-670	-25%
Tidal Flat	1934	2801	867	45%	4979	3046	158%	9689	7755	401%
Ocean Beach	363	201	-162	-45%	287	-76	-21%	470	107	30%

Estuarine Beach	22	127	105	na[1]	230	208	na[1]	302	280	na[1]
Transitional Saltmarsh	0	2488	2488	na[1]	3137	3137	na[1]	7354	7354	na[1]
Saltmarsh	0	7014	7014	na[1]	10563	10563	na[1]	15355	15355	na[1]
**Southern Big Bend**										
Undeveloped Dry Land	67217	60945	-6272	-9%	58147	-9070	-13%	47749	-19469	-29%
Coastal Forest	49873	30305	-19568	-39%	25218	-24655	-49%	15760	-34112	-68%
Developed Dry Land	45486	43340	-2146	-5%	42103	-3383	-7%	38345	-7141	-16%
Saltmarsh	31754	41471	9718	31%	48584	16831	53%	19237	-12516	-39%
Inland Freshwater Marsh	7552	7183	-369	-5%	6794	-758	-10%	5059	-2493	-33%
Tidal Flat	3870	3175	-695	-18%	5345	1475	38%	23952	20083	519%
Cypress Swamp	1521	1450	-71	-5%	1381	-140	-9%	1028	-493	-32%
Tidal Swamp	443	186	-256	-58%	90	-353	-80%	3	-440	-99%
Mangrove Forest	280	230	-49	-18%	152	-128	-46%	24	-255	-91%
Tidal Freshwater Marsh	31	29	-2	-6%	19	-12	-37%	0	-31	-100%
Ocean Beach	18	162	144	na[1]	218	200	na[1]	208	190	na[1]
Transitional Saltmarsh	1	17674	17674	na[1]	13175	13175	na[1]	15405	15405	na[1]
Estuarine Beach	1	180	180	na[1]	222	222	na[1]	244	244	na[1]
Brackish Marsh	1	110	110	na[1]	107	107	na[1]	36	36	na[1]
**Tampa Bay Study Area**										
Developed Dry Land	217,894	212,625	-5,269	-2%	208,781	-9,114	-4%	193,623	-24,271	-11%
Undeveloped Dry Land	109,902	107,158	-2,744	-3%	106,034	-3,868	-4%	102,639	-7,263	-7%
Coastal Forest	32,018	30,084	-1,935	-6%	29,605	-2,413	-8%	28,393	-3,625	-11%
Tidal Flat	25,997	17,974	-8,023	-31%	11,370	-14,627	-56%	9,842	-16,154	-62%
Inland Freshwater Marsh	12,573	12,398	-175	-1%	12,297	-276	-2%	11,965	-608	-5%
Cypress Swamp	8,388	8,359	-28	0%	8,352	-36	0%	8,300	-87	-1%
Mangrove Forest	7,054	15,635	8,581	122%	18,965	11,912	169%	17,065	10,011	142%
Saltmarsh	2,047	1,501	-546	-27%	556	-1,491	-73%	41	-2,006	-98%
Ocean Beach	1,052	1,291	239	23%	1,621	570	54%	1,393	341	32%
Tidal Creek	421	421	0	0%	421	0	0%	421	0	0%

Estuarine Beach	195	458	263	135%	556	361	185%	308	113	58%
Brackish Marsh	195	45	-150	-77%	15	-180	-92%	6	-189	-97%
Ocean Flat	109	109	-1	-1%	90	-20	-18%	90	-20	-18%
Transitional Saltmarsh	83	125	41	50%	82	-1	-2%	308	224	270%
Tidal Freshwater Marsh	43	6	-37	-86%	3	-41	-94%	1	-43	-98%
Inland Shore	11	10	-1	-8%	8	-3	-26%	8	-3	-27%
Charlotte Harbor Study Area										
Undeveloped Dry Land	162096	158403	-3693	-2%	155372	-6725	-4%	146461	-15635	-10%
Developed Dry Land	155626	152691	-2936	-2%	148262	-7365	-5%	119306	-36320	-23%
Inland Freshwater Marsh	34252	34153	-99	0%	33806	-446	-1%	32747	-1506	-4%
Mangrove Forest	23981	33270	9288	39%	38515	14534	61%	29887	5906	25%
Tidal Flat	21489	2335	-19154	-89%	987	-20502	-95%	2470	-19019	-89%
Coastal Forest	17860	14439	-3420	-19%	13524	-4336	-24%	12021	-5838	-33%
Cypress Swamp	8219	8211	-8	0%	8191	-29	0%	8016	-203	-2%
Saltmarsh	7227	7231	4	0%	6889	-338	-5%	141	-7086	-98%
Ocean Beach	756	645	-111	-15%	895	139	18%	753	-3	0%
Tidal Swamp	390	25	-365	-94%	14	-376	-96%	4	-386	-99%
Transitional Saltmarsh	0	326	326	na[1]	557	557	na[1]	925	925	na[1]
Estuarine Beach	0	200	200	na[1]	309	309	na[1]	191	191	na[1]
