# Supplementary material for: Modeled Sea Level Rise Impacts on Coastal Ecosystems at Six Major Estuaries on Florida’s Gulf Coast: Implications for Adaptation Planning
Source: PLoS One. 2015 Jul 24;10(7):e0132079. doi: 10.1371/journal.pone.0132079 (PMC4514811; doi:10.1371/journal.pone.0132079)
Supplement: S6 Table — The St. Andrews/Choctawhatchee Bay Results were provided by the Texas Chapter of The Nature Conservancy. (PDF) [file pone.0132079.s006.pdf]

S6 Table. Change in coastal wetlands ecosystems and adjacent dry land for the six study areas under 3 sea level rise scenarios with developed dry land allowed to transition. The St. Andrews/Choctawhatchee Bay Results were provided by the Texas Chapter of The Nature Conservancy.

SLR Scenario		0.7 m		1 m		2 m	
Coastal Ecosystems/ Dry Land	Initial Condition	2100-IC (ha)	Percent Change	2100-IC (ha)	Percent Change	2100-IC (ha)	Percent Change
Undeveloped Dry Land	1,172,507	-18,074	-2%	-28,448	-2%	-66,526	-6%
Developed Dry Land	495,940	-11,139	-2%	-21,319	-4%	-72,485	-15%
Coastal Forest	393,096	-56,448	-14%	-69,310	-18%	-98,922	-25%
Inland Freshwater Marsh	124,384	-4,838	-4%	-7,732	-6%	-15,063	-12%
Tidal Flat	54,581	-26,987	-49%	-25,304	-46%	4,629	8%
Saltmarsh	41,147	20,390	50%	34,260	83%	7,910	19%
Cypress Swamp	33,496	-888	-3%	-1,303	-4%	-2,484	-7%
Mangrove Forest	31,314	17,821	57%	26,318	84%	15,663	50%
Brackish Marsh	10,652	6,944	65%	6,365	60%	3,224	30%
Tidal Swamp	9,471	-3,491	-37%	-5,069	-54%	-4,144	-44%
Ocean Beach	5,204	-162	-3%	559	11%	-215	-4%
Tidal Freshwater Marsh	4,418	6,564	149%	3,594	81%	1,692	38%
Estuarine Beach	2,010	172	9%	506	25%	-27	-1%
Transitional Saltmarsh	114	31,428	27507%	25,789	22571%	36,256	31732%
Wetlands Only:	1,489,297	28,879	1.94%	29,534	1.98%	-19,087	-1.28%
