# Supplementary material for: Modeled Sea Level Rise Impacts on Coastal Ecosystems at Six Major Estuaries on Florida’s Gulf Coast: Implications for Adaptation Planning
Source: PLoS One. 2015 Jul 24;10(7):e0132079. doi: 10.1371/journal.pone.0132079 (PMC4514811; doi:10.1371/journal.pone.0132079)
Supplement: S1 Text — (PDF) [file pone.0132079.s007.pdf]

S1 Text. Detailed Methods Used to Define the Numerical Input Parameters for Each Study Area and Study Area Subsite

The collection dates for the elevation and vegetation data were obtained from the metadata associated with this spatial information. Where the underlying CLC data for any one study area (or subsite) was created from more than one source, the date from the source representing the largest area was used as the vegetation data date input value.

Direction offshore refers to the predominant direction the coast is from the study area (north, south, east or west). The tidal parameters, historic (SLR) trend and salt elevation parameters were obtained or derived from information provided on the National Oceanic and Atmospheric Administration's (NOAA) tides and currents website (http://tidesandcurrents.noaa.gov/) [1]. This website provides a graphical interface for obtaining data from current and historical NOAA gauges.

NOAA stations within the project study areas that had published tidal datums were used to obtain values for great diurnal tide (GT) and are listed in S5 Table. Historic trend values for sea level were obtained from stations that are part of the National Water Level Observation Network (NWLON) and had published SLR rates.

Salt elevation was calculated from tidal stations with 2 or 3 years of water level data available. A frequency distribution based on the 2 to 3 years of tide data was used to identify the elevation at which inundation occurred no more than once a month (i.e., the salt elevation). The ratio of the salt elevation to MHHW was then used to estimate salt elevations at stations that did not have long-term water level data.

The NAVD correction (mean tide level <MTL> - NAVD88) is the datum correction needed to align the NAVD88 elevation data with the model's internal MTL datum (i.e., the NAVD88 correction factor). This parameter can either be input as a numeric value or as a raster file that covers the entire site. One of these approaches was used at each study area. For study areas where a raster file was used to calculate the NAVD88 correction factor, Vdatum software available at vdatum.noaa.gov was used to calculate correction values. For study areas where the Vdatum approach was used (Pensacola, St. Andrews/Choctawhatchee Bays, Southern Big Bend), a regularly spaced grid of points was input into Vdatum. Valid Vdatum values that were over areas classified as water in the vegetation input were then used to create a raster of correction values with the Euclidean distance function in ArcGIS.

The erosion, sedimentation and accretion and overwash data were obtained from information contained in published reports. Where more than one value for erosion, sedimentation, accretion was available for a site (or subsite, see below) from published reports, the average of these values was used. The frequency of overwash for barrier islands of less than 500 meter width was set to either the default rate of 25 years, turned off or calculated using NOAA hurricane track data (available at csc.noaa,gov/hurricanes) for the study area.

SLAMM allows for each subsite to have its own set of numeric parameters, thus allowing for spatial variation to be modeled. In addition to subsites defined due to variations in numeric input parameters, freshwater flow sites were defined in some study areas to account for areas where a large amount of freshwater flow is present that would affect the typical vegetative response to SLR. Numeric parameters were not varied with this type of subsite. Instead, within the boundaries of a freshwater flow site, the habitat switching algorithm was altered, changing the progression of one habitat type to another due to SLR.as follows: coastal forest to tidal swamp, inland freshwater marsh to tidal freshwater marsh, and tidal swamp to tidal freshwater marsh. Due to the presence of subsites in each study area, great diurnal tide (GT) values were averaged separately within each subsite and outside of the subsites (e.g., global site) to calculate the GT parameter.

Where methods used to obtain numeric input values differed from those described above, study area specific methods are provided in the study area specific sections that follow.

Study Area Specific Methods
Below, any variations from the general methods described above to apply SLAMM at each study area are noted.

<u>Pensacola Study Area</u>
The western tip of Santa Rosa Island (about 65 ha) was missing from the DEM. This small area was filled in with licensed Interferometric Synthetic Aperture Radar (IfSAR)-derived digital terrain model (DTM) data provided by the NOAA Coastal Services Center. Subsite 2 was designated as a freshwater flow area due to the influx of freshwater from the Blackwater and Yellow Rivers. Because Pensacola Bay is strongly influenced by winds [2], salt elevation was calculated for the site using the method recommended by Jonathon Clough (pers. comm. 2011):

$$\text{Salt Elevation} = \text{X}(\text{TideRange}) + \text{W}$$

Where:
Salt Elevation = calculated estimate of the "sea water elevation" in meters above MTL;
X = unitless factor that accounts for the effect of local tide range on inundation;
W = constant effect of wind, not a function of tide range.

Neither site-specific rates for Pensacola Bay for erosion, accretion and sedimentation nor rates from proximate sites were available at the time of modeling. The values used were the same used for an earlier application of SLAMM at the site [3]. A few feature shapes were slightly modified during the editing process based on comparing the land cover to aerial photography.

<u>St. Andrews/Choctawhatchee Bays study area</u>
The Nature Conservancy's Texas Chapter previously ran SLAMM simulations of the St. Andrews/Choctawhatchee Bays study area as part of their project to identify resilience indicators at a two sites along the Gulf of Mexico coastline (also including Galveston Bay in Texas) [4]. Additional SLAMM scenarios (developed dry land protected) for this study area were run using some of the input data and parameters provided by the Texas Chapter of TNC. For

this application, the delta of the Choctawhatchee River was changed from inland fresh marsh to tidal fresh marsh.

Southern Big Bend Study Area

The vegetation layer used for the SLAMM simulation was the CLC refined with other datasets. When an entire vegetation feature was categorized as saltmarsh in the CLC and as mangrove in the NWI, the SLAMM category was assigned as mangrove. Within the extent of the CLC dataset, tidal flat features from benthic or seagrass studies replaced open water [48]. The approximately 120 ha of tidal swamp identified in the Crystal River Preserve State Park Unit Management Plan [5] were used to modify the vegetation input dataset for the study area.

For some of the most seaward marsh areas, no LiDAR-derived elevation data was available Fig. 2). For these excluded areas, 1/3 arc-second data from the USGS NED was used in SLAMM's preprocessor module to estimate change. Subsites 3 and 4 were created to encompass the portions of the study area that had no LiDAR-derived DEM data. In these subsites, the SLAMM preprocessor was used to estimate vegetation change using USGS NED 1/3 arc-second DEM data.

Tampa Bay Study Area

The Tampa Bay Estuary Program (TBEP) previously ran SLAMM simulations as part of their long-standing mission to protect and restore Tampa Bay [6]. SLAMM was re-run for this study to maintain consistency across study area using some of the DEM and vegetation data provided by TBEP (Fig. 2). The vegetation raster and DEM were resampled from a 10m to a 15m cell size due to computer hardware constraints. The elevation data (Fig. 3) used for the simulation modeling did not extend to all of the tidal flats delineated in Tampa Bay. Tidal flat areas with no elevation data were not simulated by SLAMM, and are not shown in the vegetation layer or the simulated results.

For this study area, a raster input for the MTL-NAVD88 parameter was not used. Applying the Euclidean distance function to a grid of point values from the Vdatum software resulted in outlier values being propagated over large portions of the study area. Instead, the point values were averaged by subsite, and entered as numeric parameters.

Charlotte Harbor Study Area

A few inaccuracies were identified in the CLC for the study area and were corrected as follows: areas identified by the NWI as tidal flats were used to replace the CLC classification if they overlaid water. In addition, the beach distribution data were inconsistent between the Southwest Florida Water Management District (SWFWMD) and South Florida Water Management District (SFWMD) data. The SWFWMD dataset did not show beach distribution data along the barrier island in the northern portion of the study area. To correct this, the beach information from the vegetation raster was eliminated and the FWC-FWRI beaches layer, which originated from the FWC-FWRI's 2003 Florida Vegetation and Land Cover dataset (http://www.fnai.org/gisdata.cfm) was added in.

The elevation data were downloaded as DEMs from the NOAA Coastal Services Center's Digital Coast website in the State Plane Coordinate System (Florida West 1983), with a vertical datum of NAVD88, by averaging ground points within a 5 m cell. The floating-point DEM data were converted to an ArcGIS grid format (Esri ArcGIS 9.3), re-sampled to 30 m cell size, and clipped to the study area (Fig. 3).

References

1. National Oceanic and Atmospheric Administration. Tides and currents website, sea level rise trends. Available: http://tidesandcurrents.noaa.gov/sltrends/sltrends.html. Visited 12-30-14.
2. Yates KK, Greening H. Chap 1 In Yates, K.K., Greening, Holly, and Morrison, Gerold, eds., 2011, Integrating Science and Resource Management in Tampa Bay, Florida: U.S. Geological Survey Circular 1348, 280 p.
3. Clough J. Application of SLAMM 4.1 to Nine Sites in Florida. Seattle, Washington: National Wildlife Federation; 2006.
4. Thompson R, Brenner J, Gilmer B. Informing conservation planning using future sea-level rise and storm surge modeling impact scenarios in the Northern Gulf of Mexico. Ocean Coast Manag. 2014; 100: 51-62.
5. Florida Fish and Wildlife Conservation Commission, Fish and Wildlife Research Institute. Tidal Flats Florida. 2009. Available at: http://myfwc.com/research/gis/data-maps/terrestrial/fl-vegetation-land-cover/
6. Office of Coastal and Aquatic Managed Areas, Division of Recreation and Parks. Crystal River Preserve State Park Unit Management Plan. Tallahassee, Florida: Florida Department of Environmental Protection; 2004.

7. Sherwood E, Greening H. Critical Coastal Habitat Vulnerability Assessment for the Tampa Bay Estuary: Projected Changes to Habitats due to Sea Level Rise and Climate Change. Tampa, Florida: Tampa Bay Estuary Program for EPA's Climate Ready Estuaries Program; 2013.
